# Salidroside protects RGC from pyroptosis in diabetes-induced retinopathy associated with NLRP3, NFEZL2 and NGKB1, revealed by network pharmacology analysis and experimental validation

**DOI:** 10.1186/s40001-023-01578-6

**Published:** 2024-01-20

**Authors:** Lan-Chun Zhang, Na Li, Min Xu, Ji-Lin Chen, Hua He, Jia Liu, Ting-Hua Wang, Zhong-Fu Zuo

**Affiliations:** 1 Department of Laboratory Animal Science, Institute of Neuroscience, Kunming Medical University, Kunming, 650500 China; 2 Liaoning Key Laboratory of Diabetic Cognitive and Perceptive Dysfunction, Jinzhou Medical University, Jinzhou, China; 3 Department of Anatomy, College of Basic Medicine, Jinzhou Medical University, Jinzhou, 121000 China; 4 Department of Pharmacology, Haiyuan College of Kunming Medical University, Kunming, 650106 Yunnan China

**Keywords:** Salidroside, Diabetic Retinopathy, Pyroptosis, Network Pharmacology, Molecular Docking

## Abstract

**Objective:**

To investigate the effect of salidroside (SAL) in protecting retinal ganglion cell (RGC) from pyroptosis and explore associated molecular network mechanism in diabetic retinapathy (DR) rats.

**Methods:**

HE, Nissl and immunofluorescence staining were used to observe the retinal morphological change, and the related target genes for salidroside, DR and pyroptosis were downloaded from GeneCard database. Then Venny, PPI, GO, KEGG analysis and molecular docking were used to reveal molecular network mechanism of SAL in inhibiting the pyroptosis of RGC. Lastly, all hub genes were confirmed by using qPCR.

**Results:**

HE and Nissl staining showed that SAL could improve the pathological structure known as pyroptosis in diabetic retina, and the fluorescence detection of pyroptosis marker in DM group was the strongest, while they decreased in the SAL group(*P* < 0.05)). Network pharmacological analysis showed 6 intersecting genes were obtained by venny analysis. GO and KEGG analysis showed 9 biological process, 3 molecular function and 3 signaling pathways were involved. Importantly, molecular docking showed that NFE2L2, NFKB1, NLRP3, PARK2 and SIRT1 could combine with salidroside, and qPCR validates the convincible change of CASP3, NFE2L2, NFKB1, NLRP3, PARK2 and SIRT1.

**Conclusion:**

Salidroside can significantly improve diabetes-inducedRGC pyrotosis in retina, in which, the underlying mechanism is associated with the NLRP3, NFEZL2 and NGKB1 regulation.

## Introduction

Diabetic retinopathy (DR), mainly caused by neurovascular damage of retina, is the primary cause of visual impairment, which affects the vision of patients and even leads to blindness [19]. It was reported that there were approximately 463 million DM patients worldwide and this figure was expected to increase to 700 million patients in the following 25 years [21]. Some research proved that non-proliferative diabetic retinopathy(NPDR) was present in 25% of patients in 5 years after DM diagnosis, in 60% at 10 years and 80% at 15 years [10]. On the other hand, proliferative diabetic retinopathy(PDR) was found in 2% of patients with DM duration of less than 5 years and in 15.5% of patients who had DM for 15 years or more [13, 26]. In the early stages of diabetes, it is increasingly recognized that complex neuronal, glial, and microvascular abnormalities gradually disrupt retinal function. However, the concrete underlying cellular mechanism keeps to be known.

Pyroptosis, also known as cell inflammatory necrosis, is a programmed cell death that is involved in the development of a variety of microvascular complications of diabetes [4, 14]. Studies have found that the mechanism of pyroptosis in diabetic microvasculature is mediated by the activation of inflammasomes such as NLR Family Pyrin Domain Containing 3(NLRP3) and the activation of its downstream effector Caspase-1, resulting in the release of a large number of inflammatory factors [15]. Caspase-1 promotes interleukin-1β (IL-1β), Interleukin 18 (IL-18) forms inflammatory cells and increases the inflammatory response [17]. Under pyroptosis condition, Caspase-4/5/11 binds to bacterial lipopolysaccharide and becomes activated by oligomerization [25, 36]. Activation of Caspase-4/5/11 divides Gasdermin D (GSDMD) protein, and the generated active N-terminal domain of GSDMD protein can mediate the dissolution of the cell membrane, and finally activate the NLRP3 inflammasome to activate Caspase-1, produce IL-1β, and eventually lead to cellular hypertrophy [38]. Previously, the important role of pyroptosis in the occurrence and development of DR has been well known. Therefore, it is possible to develop the effective treatment methods on traditional Chinese medicine for DR prevention.

Salidroside (SAL) an effective Chinese medicine that has anti-inflammatory [35], antioxidant [39], hypoglycemic [23] and other effects, as well as significantly hypoglycemic and neuroprotective effects, has been well known, so as that it can be used for the treatment of neurodegenerative diseases, cardiovascular diseases, diabetes, cancer and many other diseases [41]. Current treatment methods for DR, such as intravitreal injection of remizumab, retinal laser photocoagulation and vitrectomy, have no optimal therapeutic effect, with only t neuropathy to be delayed [13]. Comparatively, Salidroside can inhibit neuronal apoptosis and reduce the release of inflammatory factors, which also has a variety of pharmacological activities mild, safe and cheap, at the same time few adverse reactions, with long-term use advantage [5]. Also, Salidroside can inhibit the apoptosis of retinal pigment epithelial cells and retinal endothelial cells induced by hydrogen peroxide through the mechanism of anti-oxidative stress [27]. But the evidence on salidroside for retina glanglion cells (RGC) protection is completely unknown and the underlying pertinent gene mechanism is not clear and waiting to be elucidated.

In this study, we primarily explored the protective effect of salidroside for RGC in DR model and determine related gene changes, by network pharmacology and molecular docking [31, 40], combined with quantitative PCR validation. Our findings will further enrich the knowledge in preventing pathogenesis of DR, and provided a theoretical basis for the clinical usage of salidroside in DR treatment.

## Methods

### Preparation and administration of animal model

SD rats weighting 180 ± 200 g were purchased from the Department of Experimental Animal Science, Kunming Medical University,approved by the Animal Experiment Ethics Review Committee of Kunming Medical University, the approval number is KMMU20220894.The weight of the rats was 180–200 g. The animals were housed in a 12 h light/dark cycle at room temperature of 20–25 °C with 45% to 65% relative humidity and provided with standard food and water. After feeding for 3 days, the health status of the animals was observed and recorded. The rats were divided into 3 groups: normal control(CON) group, SAL treatment(SAL) group and diabetic model(DM) group.

After rats were weighed, STZ it was dissolved in sterile citric acid-sodium citrate buffer with a pH value of 4.5, and a concentration of 0.1 mol/L was prepared for later usage. In detail, before the model preparation, rats were fasted for 12 h and subjects to intraperitoneally injection with STZ at 65 mg/kg. After 2 h of modeling, they started to eat, then the blood of the tail vein tip was detected with a blood glucose meter after 3 days of administration, and the rats with fasting blood glucose greater than 16.7 mmol/L were used as the diabetes model, of which, the number of animals in DM group-up to 10 rats were designed as normal control.

After 6 weeks of feeding, the diabetic model group were divided into DM group (*n* = 9) and SAL administrated group (*n* = 8), except 3 rats died during the process The SAL solution was prepared with normal saline, and the rats in the treatment group were and treated by intragastric administration until 12 weeks, whereas, rats in the diabetic group were given the same amount of normal saline intragastric administration, and 2 rat died during the latter 4 weeks correspondingly, whereas,7 rats was continuously survived for 4 weeks in SAL administrated group except 1 rat died. All rats survived was carefully given nursing during all process till 3 months.

### Sample harvest

Body weight and blood glucose levels were measured every 2 weeks during the 3 month treatment period. After 3 months of treatment, the rats were sacrificed by excessive anesthesia with 200 mg/kg sodium pentobarbital. After death was confirmed by monitoring for cessation of breathing and heartbeat, eyes from each rat in all group were isolated and fixed with 4% paraformaldehyde at room temperature.

### Frozen section

After the eye tissue was taken, it was fixed in the same PFA fixative overnight, and the fixed eyeball tissue was taken and frozen in 10% (about 7 h), 20% (about 4 h) and 30% (about 12 h) sucrose solution respectively. Protection (4 °C) was kept until the tissue samples completely sink in each solution. Then we took out and absorbed the water; then cut off the excess flesh tissue around the eyeball, and injected a little sucrose into the cornea to make it more plump. Next, they were embedded with a plastic pipette Short fix with O.C.T., filled with O.C.T., at − 20 °C for 30 min, and the 3 eye tissues from each group were cut into 10 µm in coronal surface of the eyeball.

### Hematoxylin–eosin staining (HE staining)

Tissues from DM group, CON group, and SAL group were put in an oven at 37 °C for 10 min, and washed three times with PBST for 1 min each, then added hematoxylin staining solution for about 4 min, and rinsed with tap water (purple). Subsequently, blue-returning solution was added to return to blue, with quickly wash with tap water and then add differentiation solution was added, and rinsed with tap water for 5 min. Lastly, eosin was added for about 2 min, and all sections were passed 85%, 95%, 100% I, 100% II in sequence (about 1 min), and TOI, TOII was transparent for 3 min each, air-dried, sealed with neutral gum, and examined by microscopy.

### Nissl staining

Sections from DM group, CON group, and SAL group were put in an oven at 37 °C for 10 min, similar with above procedure like HE staining. Nissl staining solution (covering the tissue) was then used to stain tissue for about 3–5 min, then pour off the dyeing solution with distilled water, and quickly pass 70%, 100%, TOI and TOII transparent for 3 min each, then air dry and seal with neutral gum lastexamine under microscope.

### Immunofluorescence detection of retinal expressions of NLRP3, GSDMD, Caspase-1, IL-1β, and IL-18

After eyeball embedding (DM group, CON group, SAL group) tissues were washed with PBST for 5 times/1 min; 3% goat Serum + 0. 3% Triton-100, were used to incubate at room temperature for 3 h, then primary antibody including IL-1β(R)(1:400); IL-18(R)(1:200); GSDMD(R)(1:200);NLRP3(R)(1:400);Caspase-1(R)(1:300), were used to incubate tissue overnight at 4 °C; washed with PBS for 5 times/1 min; Next, the secondary antibody known as Goat Anti-Rabbit 488 was used at room temperature for 3 h; washed with PBS for 5 time/1 min; and observed by fluorescence microscope after mounting. Lastly, DAPI (1:3000) and anti-fluorescence and anti-fade mountant were overlap the sections to avoid fluorescent detection.

### Gene query of diabetic retinopathy, pyroptosis, salidroside

Related genes forpyroptosis, and salidroside-related were downloaded from GeneCards (GeneCards—Human Genes|Gene Database|Gene Search), after inputing their related keywords.

### Venny intersection diagram

In venny2.0.1, the three genes among diabetic retinopathy, pyroptosis and salidroside are used as the intersection genes among them. The URL is https://bioinfogp.cnb.csic.es/tools/venny/index.html [31, 40].

### GO and KEGG analysis

The intersection genes were imported into to Metascape database(https://metascape.org/gp/index.html#/main/step1) to perform GO and KEGG analysis, we selected custom analysis, then select "BP", "Enrichment" in turn CC", "MF", and "KE GG" for analysis. Export the image for analysis [31, 40].

### Protein–protein interaction and screening of Hub genes

In STRING, the website known as: https://cn.string-db.org/was used. After the interaction between genes is analyzed through cross genes, the link of multiple proteins are selected, and we performed click analysis to export the protein interaction diagram and the table of interaction relationship. The Hub gene was then screened in Cytoscape using protein interaction tables derived from PPI.

### Molecular docking verification

We used the screened core genes to query the 2D protein structure of the gene in PDB at: https://www.rcsb.org/, and searched for the chemical structure of the drug in Pubchem at: https://pubchem.ncbi.nlm.nih.gov/, for molecular docking with Autodock software.

### Real-time quantitative polymerase chain reaction(qRT-PCR)

Total RNA from the retina of three groups was extracted via using the Trizol Reagent(TaKaRa) and subject to reverse transcription via using T100TM Thermal Cycler(BIO-RAD). The RT-PCR analysis was performed in C1000 Touch TM Thermal Cycler(BIO-RAD) by using the SYBR Premix Ex TaqTM Kit (TaKaRa). The GAPDH gene was used as an endogenous control for sample normalization. All primer sequences are shown in Table 1.

**Table 1:** Primer sequences

Gene	Sense primer	Anti-sense primer
NLRP3	AAAGCAGCAGATGGAGACTGGAAAG	TGGCAGGTAGGCAGAGAAGAGG
SIRT1	CGCTGTGGCAGATTGTTATTAA	TTGATCTGAAGTCAGGAATCCC
NFE2L2	GCCTTCCTCTGCTGCCATTAGTC	TGCCTTCAGTGTGCTTCTGGTTG
NFKB1	AGGACATGGTGGTTGGCTTTGC	TCATCCGTGCTTCCAGTGTTTCG
PARK2	CCAACCTCAGACAAGGACACATCAG	TGGCGGTGGTTACATTGGAAGAC

### Statistical processing

SPSS and PS software were used for statistical analysis and graphing of the obtained data. Measurement data in each group were expressed as mean ± standard deviation (*x* ± *s*), and one-way analysis of variance was used. *P* ≤ 0.05 was considered statistically significant.

## Results

### Observation of structural changes of rat retina by HE staining and Nissl staining

**Fig. 1:**
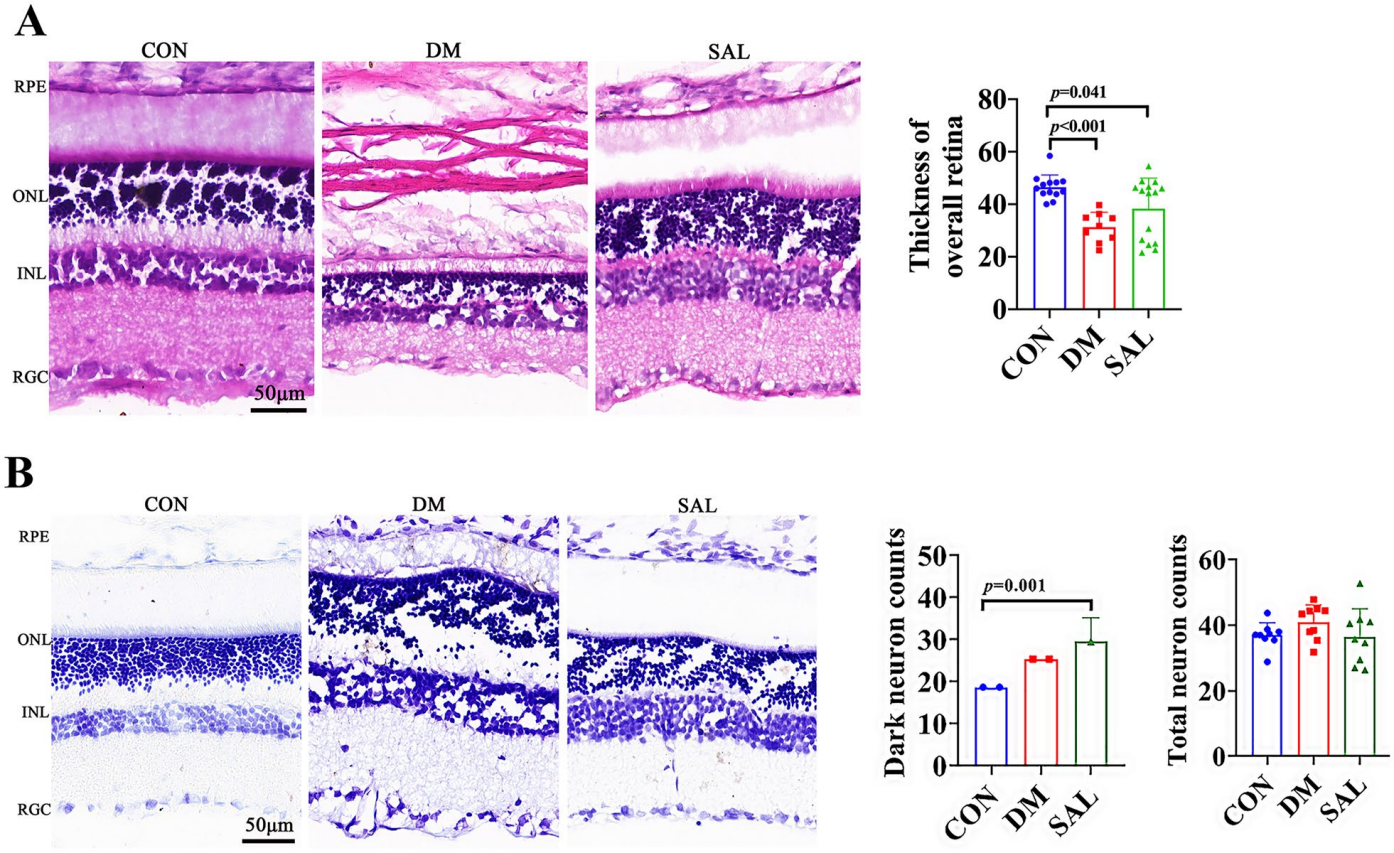
HE and Nissl staining of retinal tissue in rats with SAL treated with diabetic retinopathy. **A** HE staining of retina tissue of rats in each group and quantitative histogram of retinal thickness in each group of rats. **B** Nissl staining of retina tissue of rats in each group and quantitative histogram of total neuron counts in each group of rats. (CON: normal control group; DM: diabetic model group. RPE: retinal pigment epithelium; ONL: outer nuclear layer; INL: inner nuclear layer; RGC: retinal ganglion cell. Bar = 50 μm)

HE staining reported that the retinal layers were structurally complete and neatly arranged, with normal cell morphology, and the inner limiting membrane was clearly visible. However, the number of cells in the inner and outer nuclear layers decreased and the arrangement was sparse, in the DM group, in which, the ganglion cells were partially edema, and the blood vessel-like structures that broke through the inner and outer plexiform layers were seen. Moreover, the number of ganglion cells was reduced, and the boundary between the inner and outer nuclear layers was unclear, with disordered arrangement and vacuolar degeneration. Compared with the CON group, the retinal thickness in the DM group were definitely decreased (*P* < 0.001), while it becomes thickness in the SAL group, when compared with in the DM group (*P* < 0.001) (Fig. 1A).

Nissl staining showed that the retinal structure of the rats in the normal control group was clear, with neatly arranged. Whereas, in the DM group, the number of ganglion cells appeared obvious edema with disordered and sparse distribution. Moreover, in the SAL group, the edema of cells in each layer was significantly reduced, with relatively regular arrangement, when compared with in DM one (*P* < 0.001) (Fig. 1B).

### Immunofluorescence staining

**Fig. 2:**
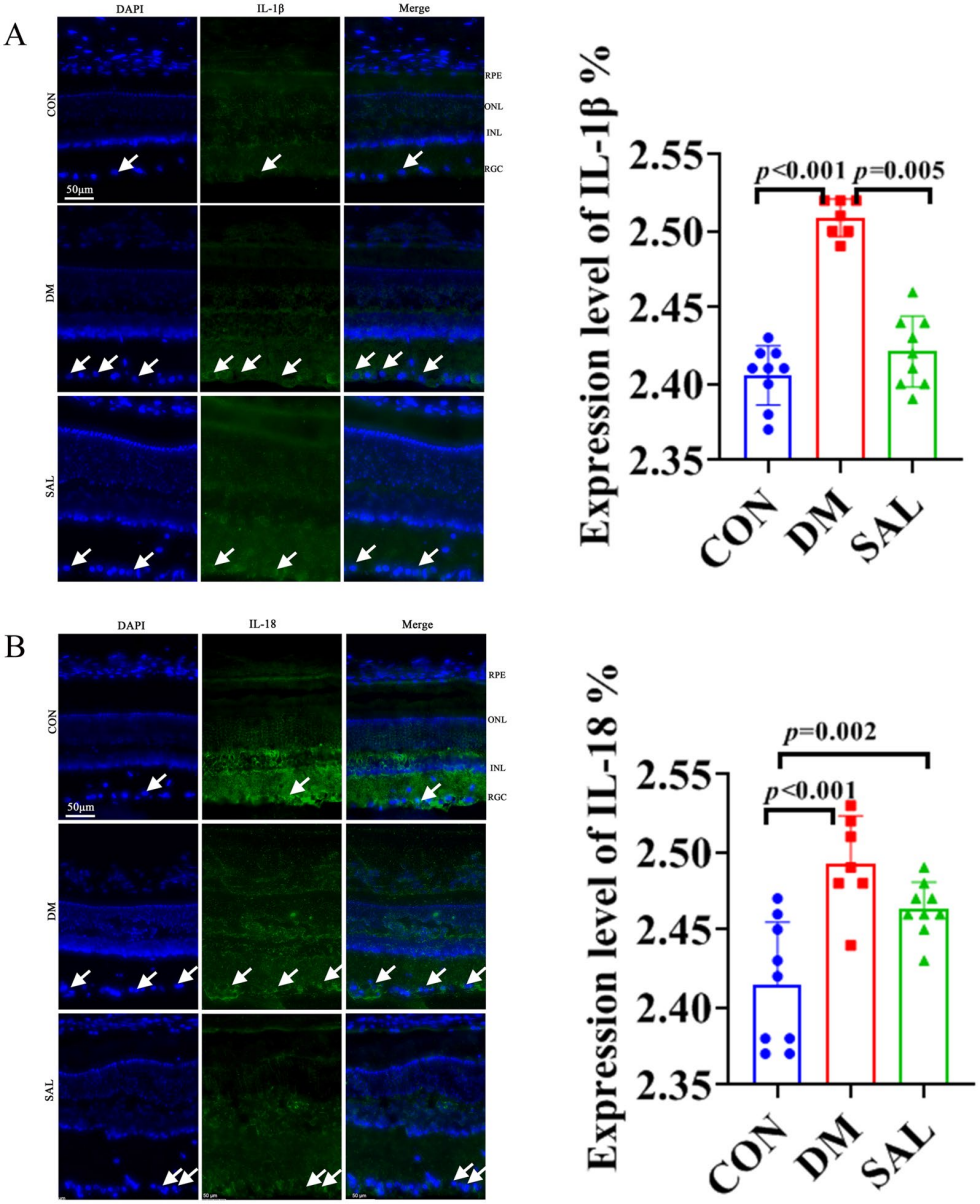
Fluorescence layout (50 µm) and statistics of CON group, DM group, and SAL group. **A** Retinal fluorescence intensity comparison of IL-1β in each group. **B** IL-18 retinal fluorescence intensity comparison in each group

**Fig. 3:**
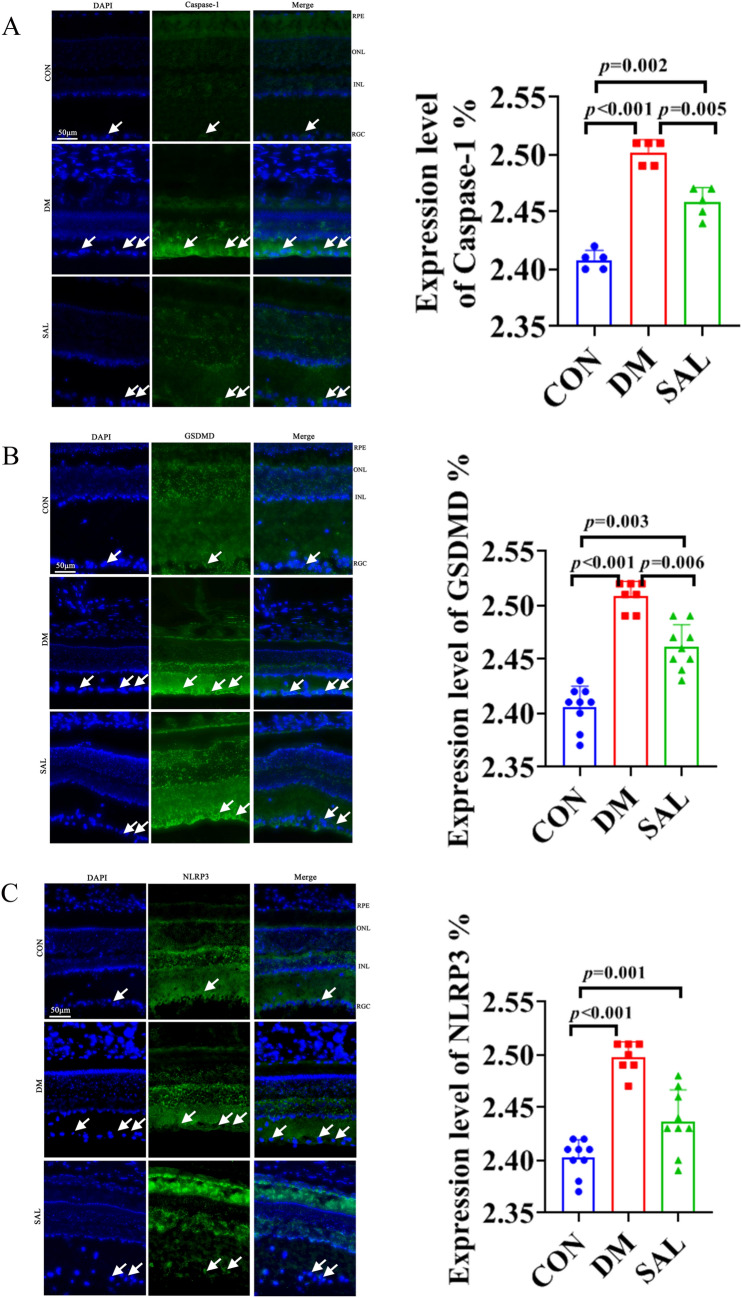
Fluorescence layout (50 µm) and statistics of CON group, DM group, and SAL group. **A** Fluorescence intensity comparison in each group for Caspase-1. **B** Retinal fluorescence intensity of GSDMD groups. **C** NLRP3 retinal fluorescence intensity in each group

The results of immunofluorescence staining showed that the five marker genes known as GSDMD, Il-18, Il-1β, NLRP3, and Caspase-1 present the fluorescence intensity in the normal group with the weakest level, but the DM group was the strongest, while it decreased in the SAL group. In detail, compared with the CON group,, the fluorescence intensity of Il-1β in the DM group got an increase (*P* < 0.001), while the fluorescence intensity of in the SAL group for Il-1β decreased significantly (Fig. 2A); For Il-18 and Caspase-1, the fluorescence intensity in DM group are also increased compared with a CON group, and they decreased in the SAL group, when compared with the DM group (Figs. 2B and 3A). The change of GSDMD is similar with above marker, its fluorescence intensity increased in the DM group (*P* < 0.001), and decreased in SAL group (Fig. 3B); Lastly, NLRP3 increased in the DM group, and decreased in SAL treatment group, also (*P* < 0.001) (Fig. 3C).

### Screening of diabetic retinopathy genes, pyroptosis genes, and salidroside genes

**Fig. 4:**
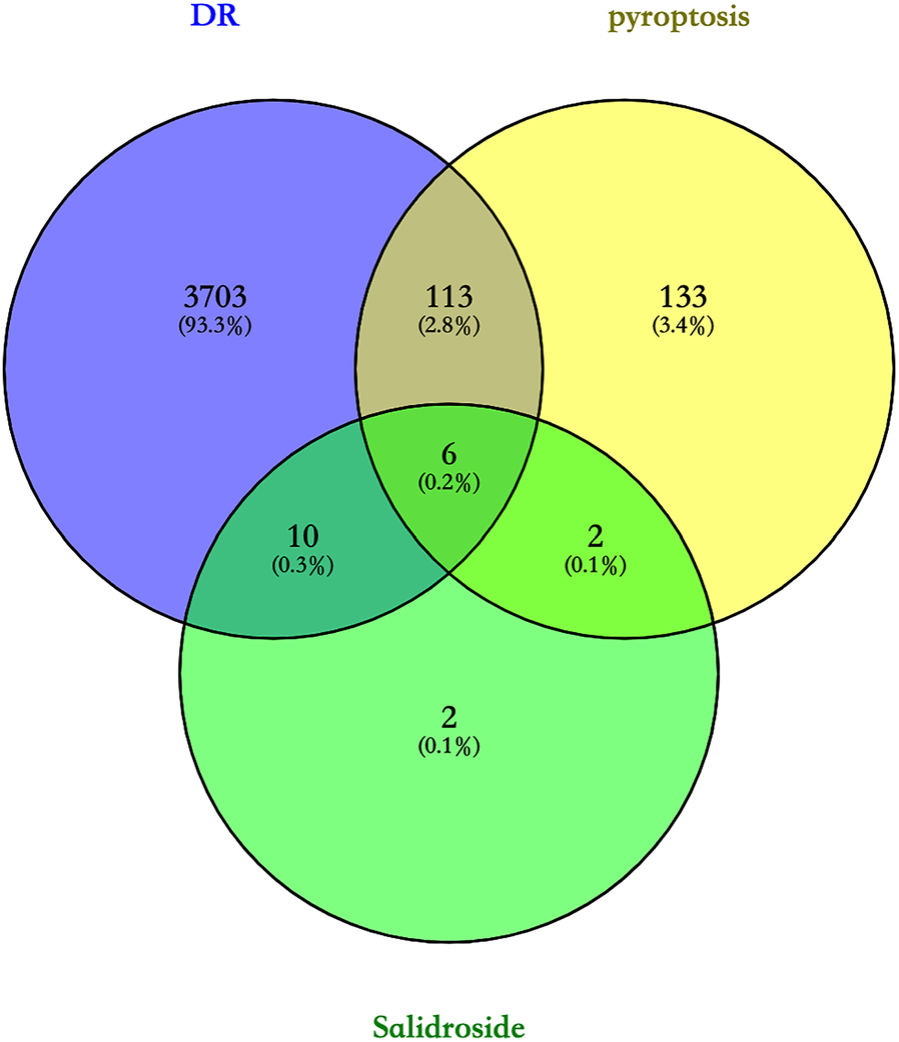
The intersection of diabetic retinopathy gene, pyroptosis gene and salidroside gene venny. Blue represents DR-related genes, yellow represents pyroptosis-related genes, and green represents salidroside-related genes

To detect the molecular network mechanism, we performed work pharmacology analysis. Firstly of all, 3832 diabetic retinopathy genes, 254 pyroptosis genes, and 20 salidroside genes were collected from Genecards. Then, they were formed cross analysis. Next, the three groups of genes were analyzed in venny2.0.1, and therewere 6 intersecting genes, namely: NFE2 Like BZIP Transcription Factor 2 (NFE2L2), Sirtuin 1 (SIRT1), Nuclear Factor Kappa B Subunit 1 (NFKB1), NLR Family Pyrin Domain Containing 3( NLRP3), Caspase 3( CASP3), Parkin RBR E3 Ubiquitin Protein Ligase(PRKN), and Fig. 4 Gene expression is shown at the end of references, also Table 2.

**Table 2:** Gene List

DR						
HSALNG0092213	INS	ITLN1	PTH	ANG	SEBOX	HRG
HSALNG0092214	GCK	PIK3CA	STAT4	BDKRB1	TYRO3	YARS2
HSALNG0042665	KCNJ11	NDUFAF2	ARL13B	KIF12	CHGB	TICAM1
HSALNG0046199	HNF1A	CYP3A4	CYP11B2	SP6	CCR7	ICAM5
HSALNG0077840	ABCC8	PDE6G	BLOC1S1-RDH5	DCTN1	FGFR2	CSF2RA
ENSG00000286361	HNF4A	EP300	WRAP53	ANGPTL2	COQ5	SRD5A3
LOC105379011	INSR	FOXO1	TDP2	CORT	CPT1C	KLHL9
L13714-196	HNF1B	SEMA4A	KCNV2	SLC6A2	H2BC21	TRIM8
RF00004-026	PDX1	MLXIPL	PEX2	NID1	ADRA1B	EPG5
piR-50443–131	WFS1	IFT88	C2CD4A	IGFBP4	SQLE	BRF2
piR-50443–400	PPARG	CDHR1	FCGR3B	UBC	NNT	PRDX4
piR-43325–031	IL6	CDC123	MIP	CXCL5	NKX2-1	HMGN1
KR153194-133	NEUROD1	LRP6	SMAD3	ERAP1	TIAM1	GNE
HSALNG0008511	VEGFA	TEK	CYCS	MIR24-1	IDH2	VGF
ENSG00000270981	PAX4	CHI3L1	NGFR	COQ9	REPS1	TAX1BP3
LOC105375610	TCF7L2	PMM2	FOXM1	KCNMB1	GTPBP3	P2RX5-TAX1BP3
piR-52680–287	ACE	GLI2	NDUFAF1	TERF2IP	TCF19	CA14
piR-58297–391	IRS1	HADHB	DLG4	SAR1B	HDAC6	CERK
LOC107986902	AVPR2	PRPF6	AQP4	WDR72	SLC1A3	MIR133B
TPCN2	SLC30A8	BCL2	HRAS	COL4A2	GNA11	SMARCB1
CNTROB	RETN	KLHL7	H19	NTN1	RTTN	AKT1S1
ALDH6A1	SLC2A2	BRCA2	PLA2G5	CDC42	TERF2	TNNC2
PINLYP	AVP	PCK2	KCNK16	PIGF	RGS9	LDLRAP1
BLOC1S3	KLF11	MT-TQ	TXN	PML	PUS1	LSS
RNU105C	ENPP1	PRSS1	STAT5B	ACADVL	IFT20	CHRD
CALCRL	INS-IGF2	CNGB3	IL23R	COL11A1	ITGAX	LIG3
NME2	IGF2BP2	MMP1	AXL	RAB8A	TUBB	HCAR2
CD2	PON1	C3	CLCC1	NSD1	FOLH1	ANKH
NEIL1	HFE	PCSK9	RTEL1	MPRIP	EFEMP2	HCP5
ABCD3	AKT2	DHDDS	CD8A	PDSS1	ABO	PLEKHB1
ADGRG6	BLK	TF	MIR25	HS6ST3	MAEA	ODC1
LAMA1	PTPN22	CALCA	CACNA1C	SLC2A10	TRPC3	MIR202
FAH	IRS2	PRPF4	BAD	OCLN	MTHFS	COBL
SNX14	GLIS3	APOC2	TBX18	VSTM2B	SEMA3E	SERPINB5
RAD23A	EPO	CACNA1A	ARMS2	MAP2K1	U2AF1	MID1
PGLYRP1	MTNR1B	FGF8	ERBB3	DNAJB11	LECT2	TRAP1
SAMM50	LMNA	SOCS1	SGSH	TTC26	HES7	GRK5
PPRC1	MT-TL1	CD163	ACAD9	KLK1	KHK	IFNG-AS1
HIF3A	CEL	PDCD1	PRDX1	VEGFD	TCTN2	FAM3B
PANK3	ADIPOQ	HK2	MTHFD1	ABCB11	TCIRG1	LY86-AS1
CUL4B	ALMS1	HSPA5	BCS1L	CREB5	UTS2R	MIR125B1
TCEA1	ZFP57	PPY	FABP12	CCDC68	ETFB	NCF2
GJA8	TBC1D4	ANXA5	CYBB	MRPS15	FAM135A	EXOC4
ZEB1-AS1	AKR1B1	CLU	RS1	OSCP1	XIST	SLC16A7
TUBG1	TNF	ONECUT1	KDM4C	MAP3K21	C5orf67	MYO5B
EPB41	PTPN1	PIK3C2A	ATF4	LINC00917	BANF1	ZIC1
ADAMTS18	ALB	PRKAB1	SAMHD1	LINC02774	LNX1	SLC22A6
LEFTY2	SOD2	MGAM	IFNGR1	LOC100506023	LBR	TRAF3IP2
MYO3A	FOXP3	HSD11B1	MECP2	LOC101928236	KCTD7	MFGE8
ETV4	CTLA4	MT-TS1	NDUFS3	LOC729200	UBE2I	PTPRF
EDEM1	HLA-DQB1	FGF23	NDUFB11	LOC100131080	LOC109113863	XPC
GNGT1	HMGA1	GAPDH	NDUFAF5	HSPA1B	HDAC4	PLXND1
KIFAP3	MT-TE	TIMP2	MPZ	HCFC1	SCARA5	CYP1B1
EXOSC3	CAPN10	PYY	SDC3	CEP162	MDC1	CTBP2
DNTT	MIR29A	CYP2C19	SI	DNMT1	LOC110973015	IQGAP1
CNOT3	APPL1	MIR34A	NKX2-5	AGMO	CUL3	RAMP2
GABARAPL1	DCAF17	SERPINA12	GZMB	CD46	CSNK2B	STC1
EML1	AGER	APOM	LOX	MMP10	C4B	LGALS3BP
C2CD3	NOS3	TGFB2	CLTRN	CYP2C8	PARP2	EHD1
POLR3K	IGF1	CNOT1	AQP5	COL8A1	PENK	PLD2
RBPMS	LEP	MAK	HSP90AA1	COL11A2	MSH2	GRM6
ASAP1	CRP	CCK	HOTAIR	LPP	RPA1	KPNA1
EXOSC8	CCL2	MT-TF	COQ2	ISG15	TMCO1	USH1G
CNGA2	ICAM1	ST3GAL4	GREM1	PPIG	MYCN	PDZD7
CNNM4	HLA-DRB1	CD34	SDHAF1	IREB2	TFEB	ESPN
TMC1	MIR29C	HSD11B2	CD44	G6PC3	ZP4	OPN1LW
NME8	HLA-DQA1	CLN3	CCL11	VPS13A	CSF1R	PRPF38A
PPIH	MTHFR	HSPD1	SCO2	LARS2	INPP4B	WDR17
SRPX	RHO	NEK2	POLG2	VIM-AS1	PER1	CYP2U1
DNAI2	MC4R	SLC7A14	GBA	NDUFA10	MPP4	CWC27
CIB2	APOE	GC	CCR6	RGS9BP	COL4A3	PITPNM3
TGOLN2	PTF1A	FTH1	IL5	DCDC2	TUBD1	AAR2
ABHD2	IL2RA	OXT	BTC	SNHG18	OXA1L	MIR7-1
LRIT3	PLAGL1	NR3C1	RNASEH2A	TBCCD1	SPTA1	ENGASE
VSX1	RFX6	TCF4	HPSE	TRPS1	FN3KRP	FRMD6
LHX2	GCG	SIM1	DNM1L	MCHR1	ATF6B	APELA
HELQ	MIR21	CCDC28B	SLC25A4	GYPB	ARF6	TIE1
MYCL	ABCA4	CASP3	TRPV4	PDGFA	ASPA	M6PR
GPR179	HFE-AS1	KRTCAP3	TTPA	DDX39B	UCHL1	SRRT
POU4F2	IL1RN	TGIF1	ELOVL4	TTC21B	ART1	UST
RAB3IP	HYMAI	ATF6	PRKCA	CDH13	CDC42EP3	FSTL5
RSPH4A	MT-TK	CPT2	AXIN1	TXNRD2	SUV39H2	TBC1D5
U2AF2	EIF2AK3	ACHE	TUG1	MIR103A1	ARSB	SEC11C
KIAA0586	STAT3	PTPN2	MEG8	CDK2	KCNQ5	HCG27
RRH	CAT	RDH5	ACD	NDUFA4	TNFSF18	RPS15AP30
TUT1	LEPR	VTN	MIR216A	SLC7A14-AS1	C6orf47	RN7SL865P
CEP83	BBS2	SIX3	TMEM231	MC1R	SMPD1	HSALNG0080722
EGFLAM	ITPR3	IFT74	CD1A	NUDT6	ASCL1	DDX23
DNAAF2	SERPINE1	CFAP418-AS1	GLA	CDKN1B	CHD7	ZNF644
LRIT1	EDN1	SHC1	TSC2	SLC9A1	TPX2	RHBDD2
STRC	CTNNB1	KRT18	FGF1	IL3	ITGAV	TENT5A
TMC2	SLC2A1	ACACB	CCL4	ADAM17	MIR183	F13B
CRYZL1	IAPP	ISL1	NAA15	BIRC5	PCDH18	SERPIND1
ABHD16A	SUMO4	ARL2BP	DDHD2	SETDB1	GSDMC	LLGL2
NXNL2	IL1B	KCNJ10	CASP14	GLB1	OR12D3	PTK2
OR2W3	CDKAL1	LIPG	NPHP3	ATG7	IFT81	MIPEP
PTPRQ	SAG	KRAS	POSTN	MTR	CEP295	OPLAH
SPP2	LRP5	PNPLA3	PCDH15	LCT	AGK	MPP2
VEZT	FLT1	IMPG1	SLIT2	FEN1	GUCY2F	DISC1
FBXO45	GAD2	IL15	APOH	GNAT2	A2M	KCNA1
OPN1MW	APOB	IL6-AS1	MIR127	DDC	SMN1	RORC
SAMD11	MT-ATP6	CPT1A	MIR9-1	WG	HEPH	SLC22A12
SAMD7	GATA6	XBP1	IMMT	NRXN3	TCEA3	VKORC1
DNAJC14	MT-ND1	TFRC	ENSA	CHPT1	ALDH7A1	RNF216
LRIT2	REN	TAP2	RAF1	ELAVL1	MPDZ	PPP1R12A
PRSS56	AGTR1	NODAL	CXCR3	TERF1	BCL9	FARS2
FRYL	TGFB1	TSPAN8	CYP24A1	ABCB7	RABAC1	SLC25A24
IGSF9B	DNAJC3	UMOD	GCLC	AP5Z1	TUBA1A	ORMDL3
PYDC1	PTPRN	LGALS3	SNCA	TRPC6	CHKB	EHMT2
VSIR	CDKN2A	POLD1	FH	ALK	LOC110008580	CLDN5
ZNF596	ADRB3	STX1A	MDM2	MAF	FANCM	SLC10A2
OR5K2	CAV1	HADH	NKX6-2	IL18R1	TBX4	DNAH11
KRCC1	BEST1	MYH9	GRP	HBG2	CSNK2A1	KCNC3
CFAP36	MLKL	SCT	ABCB1	MIOX	GMPPA	FGF13
TDRP	VWF	F8	TRPM1	GPX4	MRRF	SLC45A3
DNAAF6	CCR5	TG	BCL11A	CNTLN	GPHN	TARDBP
FAM227B	IL10	MIR20A	CCR2	IL10RA	IMPDH2	LPCAT1
FAM218A	SST	IL1A	TNFRSF10A	MKI67	PROCR	ELOA
C10orf105	BBS1	TNFSF11	ERBB2	ACKR1	HELZ	ARRB2
OR56A5	NOS2	PTPN3	NR2F2	PC	SRSF6	GNGT2
DIO3OS	CP	USH1C	RD3	SLC25A11	MRC1	MIRLET7D
GOLGA8S	UCP3	H6PD	RNASEH2B	LRRK2	NOD1	MBNL1
SMIM43	AGT	CBS	RBP1	CDKN1A	C9orf72	DDB2
HOTAIRM1	MIR17	SCARB1	VPS33B	MIR9-3	PRKD1	DECR1
RNU11	MIR155	GJA1	JAK2	SCO1	RECQL5	KIFC3
PCBP1-AS1	RPE65	ARAP1	ALDOB	GDF1	FAF1	ZBTB24
LINC00877	SERPINF1	CYP4V2	KLF4	PEX3	NTF4	KCNIP1
RNU6ATAC	PIK3R1	FOXH1	PIK3CD	APEX1	DPYSL5	TMEM126A
ENTPD1-AS1	TULP1	CASP9	CXCL9	PDE2A	ALMS1P1	STAT6
MIR1262	PRKCB	CNDP1	CTSB	SPG7	ACAT1	HSD17B8
RN7SL3	APOA1	PDGFB	MEFV	COX6B1	EFNA1	AMELX
LINC00570	FGF2	F5	BAX	PDGFRA	KHSRP	CCL18
CHRM3-AS2	SORD	TPO	HPX	MMP7	CEP78	CCDC86
MEIS1-AS2	MIR145	FASN	IL12A	DNAJB1	P4HB	CD247
LINC00379	ANGPT2	STK11	NDUFA13	NFAT5	LCK	LILRB1
LINC01589	TLR4	CYP2D6	IL7	PLXDC2	PDC	LILRB3
LAMA5-AS1	LPL	PROS1	GP1BA	KIAA0825	SUOX	GGT2P
CATIP-AS1	USH2A	KLF14	ADCY10	MIR211	BIRC3	SGCB
SMIM27	AKT1	C1QTNF9	NDUFAF3	CARD14	H3-7	RRM1
CDH23-AS1	UCP2	SOST	MAPK3	FCGR3A	ARAP2	TRAPPC3
LINC01571	C12orf43	TAP1	AQP1	MAN2A1	LYRM4	DNMT3B
NDUFA6-DT	VCAM1	FAS	NAT2	SSTR2	GCNT1	CUL5
LRRC8C-DT	VDR	MAPK1	SUCNR1	SYK	B9D1	PEX19
MELTF-AS1	PRPH2	MBL2	SMC1A	CD86	WDR35	SMCHD1
MIR4494	IGFBP3	CACNG2-DT	ZFAND3	PLVAP	IFIT1	GJB6
PURPL	ARL6	DDIT3	ECHS1	CD80	NR4A2	MYLK
KIAA1671-AS1	AIRE	MMP8	ATL1	NGLY1	TMEM70	RPTOR
UBE2R2-AS1	FTO	C1QTNF3	DKC1	PPP2R2B	SMN2	SMO
FLVCR2-AS1	GAD1	OGG1	NHP2	SORL1	B4GALT2	CDH2
ATP2C2-AS1	TTC8	PDP1	NOP10	ADORA2A	CD96	LOC116158507
ETV5-AS1	BBS4	SLC30A10	FOXA1	ZNF23	ASIC5	ZFPM1
LINC01933	RLBP1	GAREM2	DNAH8	CASP7	TLR6	LOC118142757
LINC02470	CISD2	FASLG	NEU1	ALAS2	FCGR2B	VNN1
OSMR-DT	MMP9	PDE5A	NPY2R	MRPL44	SCARB2	SMARCA5
CEP83-DT	KDR	PNLIP	ATP5MK	ARNT	PDE6D	MT-TM
LOC339685	HIF1A	LARGE1	ACADM	BPI	LIN28A	CAST
CARMAL	DPP4	ENO1	KRT8	TMEM216	TTC39C	STK4
LINC02605	CEP290	GSTT1	CCNL1	NELFA	POC5	ALG12
PP12613	TP53	GDNF	L1CAM	XK	NDUFB6	MDH1
RNU12-2P	GLP1R	SCD	TGFA	UCHL3	DZANK1	H3C14
AGAP14P	SOD1	CD79A	LINC00343	COMMD6	DGKQ	AGXT2
LINC03000	SELE	DDAH2	GSS	CCR3	ROMO1	UQCRC2
LOC107986845	RBP4	CS	XYLT2	PIEZO1	PSMB10	RMI1
ENSG00000254564	SCAPER	MT-TY	P2RX7	VPS11	UGT1A6	MUTYH
LOC112841608	BBIP1	ENO2	IL7R	ABCC4	MIR29B2	FLOT1
OPSIN-LCR	AOC3	MT-ND2	APOL1	C1QTNF6	UVSSA	RNF145
RDPA	MEN1	NOX1	SLC19A1	PDE6C	TYMS	UBTF
DFCTRPS	HAMP	PHF3	CA1	SPG11	UGT1A8	RCC1
RPY	GHRL	ALOX5	PAGR1	MMP12	EIF2B2	LIN7A
PTPN6	IGFBP1	GATA3	CLDN4	BECN1	SOAT1	GDPD1
ALG14	BDNF	PLG	BSG	SLC26A4	DDIT4	SMAP1
TRIM50	HP	MMP14	KRBOX4	MT2A	GNAI1	CRB3
IL2RG	CRB1	TLR9	CCDC144NL	POLR2A	GADL1	KLHL31
SLC25A38	NEUROG3	PAX6	MIR1179	XRCC6	RORB	ZC3H8
HPGDS	NR2E3	SERPINA1	KITLG	SLC4A1	MTO1	LGSN
ATAD3B	BBS10	ANGPTL3	CSF1	ST6GAL1	DBH	FAM161B
SPTAN1	AR	C1QTNF5	MGMT	EZR	IL37	YPEL2
ERVW-1	GCGR	CHM	COX10	NUBPL	UGGT2	OGFRL1
PLAC8	CDKN2B	ELANE	CTBP1	MIR26A2	GRIN2B	ZBTB8B
MYO18A	TNFRSF11B	MT-ND3	THY1	CEP164	SIX6	TTLL10
PIWIL1	IFIH1	ABCG1	TRPM3	CALB1	SMC3	TENT5D
FBXW5	MMP2	NCF1	MME	C12orf29	GATM	UMAD1
PDGFD	IMPG2	CDH5	XYLT1	GRK1	NCALD	RP9P
LAMA5	CXCL8	MT-TV	MIR205	MDH2	NT5E	FAM225A
LINC00871	IGF2	POLR1C	MIR142	IGFBP6	IFT22	ITGA6-AS1
HEXA	BBS7	LHX1	NEK9	GRK7	ABHD6	LINC00940
TIMD4	MIR192	NTF3	SLC9A3	IL12RB1	ALPL	SDCBP2-AS1
RARS1	FZD4	GLO1	HSPB1	NDUFS6	TBCC	LINC01476
LAG3	MIR126	ENHO	GAS6	NDUFA6	TRIM25	LINC01564
GRIN3A	MT-CO1	NPHS1	FANCC	INVS	IRF1	GRPEL2-AS1
SLC66A1	ADRB2	PHYH	CCN1	TIRAP	IFNAR1	INKA2-AS1
ACO2	CXCL12	MAPK10	ABCG2	SPTBN5	RXRB	SYP-AS1
COX4I2	PPARGC1A	PLIN2	VIM	MPV17	NPC2	LINC01399
FSD2	MKS1	MET	C1QTNF3-AMACR	IL1RAPL2	JAM3	LINC01324
H4-16	IL18	ATP2A2	POMT2	SFTA3	RAB28	ELN-AS1
RAB27A	HHEX	PRKAB2	S100A9	TBP	ARF4	TNS2-AS1
PHGDH	RPGR	SP1	ROBO4	HLA-DOA	MESP2	ACBD3-AS1
KLC1	CCN2	TSC1	VIP	CACNA1E	PRPSAP1	MIR4263
RPIA	PPARA	NKX6-1	FXYD2	HAGH	PALM2	LINC02600
CLN6	PROM1	BRCA1	CLASP1	SLC37A4	FUCA1	LINC01297
NFATC3	CFAP418	CYP1A2	CMA1	KIF3A	SERPINE2	IGHV4-38–2
ZIC3	IGF1R	DLL1	ADCYAP1	IL22	CD19	LINC02631
GABARAP	POLG	ADRB1	KIT	ESM1	SLC27A5	RP32
MAPK9	RDH12	ZIC2	IFNA2	NUS1	MYO1A	LOC100421404
PCM1	PGF	NPC1	EDNRB	MIR124-1HG	ATPAF2	WNT1
MIR19B1	NDP	TLR2	IRF3	MIR199A2	ARL17A	MAP2K2
GUCA2A	HBA1	RPS6KB1	LOC111365141	SCGB1A1	PHOX2A	PSMC5
TSFM	CFTR	ADAMTS13	PIK3CB	CACNA1F	MAG	CA6
ADI1	R3HDML-AS1	MT-TL2	MIR217	MCL1	RCC1L	KCND2
OPTC	NAGLU	MYD88	TRPM8	SOS1	TNFAIP8	DUX4
PCCB	WRN	ITGA2	COL1A1	ETFDH	LGALS4	DUX4L1
ATP2B2	IFT172	MT-CYB	MAFB	PER2	CXCR2	TOR1A
HDAC8	ROM1	DRD2	S100A8	SLC18A2	CCL21	NLRC4
TXK	MTTP	PTPRC	ATXN10	HULC	VDAC1	MMAB
FGFRL1	EYS	OTX2	SLC6A6	VLDLR	CDK1	TRPM2
RPS6KA6	ADM	MIR195	FLT4	MIR335	IFT57	EPHA1
NAGS	CETP	PBX1	CSH1	NCAM1	SIGMAR1	H1-0
ARHGAP6	GHR	ADA2	HBA2	RIMS1	MIR542	SCN8A
STK19	HLA-B	SRC	APLNR	ATXN3	GABRR2	SLC25A17
PTCD3	KCNQ1	RPS27A	AIFM1	ALDH18A1	MIR328	CPSF6
SSPN	CRX	IL12B	PEX5	CETN2	HDAC2	OXGR1
CABYR	MYO7A	EDNRA	CISH	KIR3DL1	TBX1	MTAP
INA	LRRC32	CYP21A2	ACTB	CNDP2	RAB6A	PDPK1
EGLN1	HMOX1	REG1A	MIR27B	ABCC2	ROCK1	RANBP17
TRIM21	POMC	GAS5	UFM1	CD69	SNHG7	HBE1
TJP2	GCKR	ICOSLG	CDK5RAP3	TGFBR3	GRHPR	GUK1
WARS2	PLAT	PPT1	DDRGK1	MIR148A	IL32	MFAP4
TRADD	IFNG	LBP	UFL1	XAB2	ARL2	PHEX
CFL1	PANK2	CD4	IL4R	COA3	EWSR1	S100A6
PDIA3	MKKS	RP6	SERPINB7	C1QA	SETD7	GRIP1
SSBP3	CASR	CYGB	ZPR1	CRNDE	ARRB1	AGO1
TEN1	NAMPT	HK1	CXCR4	MAOB	CACNB2	LTB
RRM2	UCP1	GNAT1	MIA2	HBS1L	MAN2B1	CCDC26
SEC31B	MSTO1	PSMD9	PMP22	NSD2	PSMC6	MIR296
TOP3A	CXCL10	NOTCH3	CTSH	ALDH3A2	VPS4A	FLVCR2
SFXN4	APOC3	NDUFS4	PLXDC1	MTERF1	GJB1	DNAI1
SMARCA1	CST3	VEGFC	CDH3	HSPA8	GAN	DNAH5
PLD1	ATM	RET	TBL1X	RBX1	XRCC3	CRYBB2
ACOT8	FBN1	USH2A-AS2	RNASEH2C	LYVE1	KIF1A	CRYBA1
NF2	NRL	RPGRIP1L	TSPAN1	PBX2	SEC63	OTOA
HSD17B13	MT-ND4	SDHA	PRKN	THRB	RAB11A	RSPH9
SPAST	RBP3	SLC19A3	MT-RNR1	UNC119B	MMUT	CHML
TUBA4A	MIR140	ERCC2	RBFOX1	EXO1	INPP1	S1PR1
CD81	HGF	RUNX2	CCR1	FAAH	CDK5RAP1	MMP25
EXOC7	PCBD1	HLA-DPA1	FGB	VASH1	SRSF2	TFF1
NFATC4	TH	NDUFS2	LEKR1	ETV6	GRIA1	CYP26A1
COX7C	GH1	CNTF	SLC11A2	ABCC1	DCTN2	KCNK6
HEY1	PARP1	IGFBP7	ADAMTSL1	OSM	SLC2A6	GNA15
GNAZ	TIMP1	PF4	CSH2	CENPA	TPM3	NDUFA3
ASPM	THBD	H2AC18	IRF8	DGKE	SNX17	EML3
PREB	PNPLA6	NR3C2	OPA1	APTX	MRPS34	KLHL42
AKR1D1	HBB	DYNC2H1	SEMA3A	KIR2DL3	COMMD1	TMEM132D
CACNA1G	CD40LG	LOC101927157	MFRP	IL6ST	ELAC2	SLK
CD93	PTEN	TRAF6	SDHC	PDGFC	GOLIM4	STX11
HSP90AB1	GSTM1	MIR15A	TBX21	KIR2DL1	H1-2	UTRN
NR2C2	MIR377	EGFR	TXN2	MOK	CACNA1S	GPR108
ACTG2	CTNS	POLR3A	PGBD3	USP7	PCSK1N	TIPARP
SIRPA	IL4	LDLR	COASY	IFT80	MAP1LC3A	YY1
PABPC1	SLC5A2	SMAD4	CR1	WHRN	CORO2B	TNNT3
KAT2B	G6PC2	MIR144	DLL4	GYPA	PLEK	PGAP1
JRKL	GGT1	PEX1	GPR174	ACAD10	ACAA1	LFNG
KRT14	SHBG	CDON	CLDN3	PDE6H	NPAS3	TRIM16
PMPCA	LTA	NRP1	ARHGAP22	NDUFA11	MLH1	UBXN11
RARS2	NPPB	OPN4	CEP120	GLI1	GNRHR	TANC1
TRIT1	NPPA	SIRT6	SERPINI1	AHSP	GRIA4	ARL4D
HNRNPK	FGFR1	SLC24A1	ERF	SVOPL	TNFSF13B	ALDH1B1
RPS6KA3	HLA-DPB1	BMP4	TSC22D1	COPS5	SUCLA2	B4GALNT1
PAK1	NPY	AP4B1-AS1	HPE1	FCGRT	GPATCH2	RICTOR
CCL19	RP2	CASP1	ADH1B	EFNB2	NRM	PLS1
KCND3	PLA2G6	ELMO2	DNASE1	ITGAL	DAB1	TCN2
STAP1	SDHB	TKT	CD55	DLC1	TM6SF2	TPM2
VPS45	NFE2L2	CYP17A1	CUBN	CRB2	SLC15A2	TNNI2
HS2ST1	BGLAP	OGA	FTL	HAVCR2	MYOC	PNMT
PRDM9	PON2	JUN	SSTR3	PLA2G4A	TVP23B	PEX14
CFHR2	BBS9	CACNA2D4	ITGB1	AMPH	SEMA3G	MMADHC
BSPRY	PNPLA2	SDHD	IDUA	TRPC1	SDR9C7	PCSK6
FSD1L	IL17A	STYX	NDUFAF6	ASIP	ABCF1	GNAQ
SPATA3	MIA3	TGFBI	CLCN3	HABP2	MTHFSD	SACS
TRIM61	F2	TNFRSF25	MISP	EEF1A1	TSPAN2	CAPZB
PEX26	APLN	ERCC3	DRAM2	TMPRSS2	GGCT	CUL4A
AKR1C2	MT-CO2	PDE11A	IGSF21	FANCD2	F2R	SNRPB
AFF2	HLA-A	MIR210	MIR182	RYR1	AZU1	B3GAT3
DCAF1	SIRT1	MIR200B	EIF2S1	PIGG	RHEB	CCNO
SEC61A1	CD36	MT-TN	PCDH12	CYP7B1	IKBKG	SRSF1
CDS2	BBS12	SETX	CREBBP	MIR92A1	KCNJ16	AOC2
ITIH4	SPATA7	LOC105371046	BMP3	PDSS2	MYH1	IFT122
MPP3	RRM2B	ADAM9	XRCC1	MCTP2	LINC00523	EXOSC9
RPS26	TIMP3	MLN	MIR106B	SOX2-OT	RPL3P2	MYO15A
KMT5C	MIR122	TMEM67	AQP12A	ARSG	FIZ1	UXT
PRNP	MIR146A	PSMB8	PDHA1	DHCR7	NXNL1	TFPT
TMEM147	FABP2	ITGA2B	NNAT	MIR320A	SCLT1	DACT2
AGPS	TINF2	ADA	BDKRB2	SSBP1	MTRFR	PPP1R12C
WNT3A	AHI1	CRH	CRAT	HMGCL	NFATC1	TARM1
POLE2	ACP1	LRP1	ATP13A2	FKRP	FBN2	ZNF805
PDPN	SELP	ABCB4	IFNA1	DICER1	PDE4A	CFAP92
KAT5	HMGCR	COL2A1	IFT43	CTSL	COQ6	SYT9-AS1
TEAD4	HMGB1	ITGB3	KCNJ3	FBLN1	ARRDC4	LOC101928994
HNRNPA2B1	MT-ND5	APC	LOC108251801	FCN3	TBCD	RPS6KA1
TPI1	ESR1	LMNB2	AGXT	SUMO1	LINC-ROR	SEMA6A
MAP4K2	CEP19	PRTN3	UBR1	MT-RNR2	SLC4A5	PPP2R3C
ZNF570	PRPF31	C1QTNF1	TGFBR2	IGFBP5	FMR1-AS1	H4C1
F2RL2	SURF1	ITGAM	RECK	CARM1	LRP2BP	FCGR1A
APRT	TRIM32	JAG1	GCC1	CCR4	EHMT1	MCU
MYO9A	MT-ND6	ERCC1	ZNHIT3	SMAD1	SHPK	PEX11A
HIGD2B	ABCA1	ATXN7	PANK1	ANPEP	SOX9	SOX11
ATP2B4	MPO	PIK3CG	SERPINF2	NDUFB9	DDOST	LIN28B
MYH10	TREX1	DISP1	DNASE1L3	NDUFB10	GFM2	GFRA2
MYH14	BBS5	TWNK	DNMT3A	NDUFAF4	LAMB2	MYH3
CASP4	GPT	ABCG8	TNFSF4	TMEM126B	SLMAP	MYH8
TMEM176A	PDE6A	CTC1	ETS1	TIMMDC1	NOP56	CLCN5
HHIP	DLK1	CCL3	NOTCH4	NDUFAF8	SMAD9	LYPD3
HPS5	ADIPOR1	PRKAG2	PLA2G1B	SPTBN2	ETV1	C13orf42
HEY2	ANGPTL4	CA2	TBK1	IDO1	NCR3	LCN1
PPM1D	SDCCAG8	LOC107133510	DACT1	GSN	TYRP1	TNFAIP8L2
TPT1	ICA1	HARS1	HBEGF	ANK1	CCL1	MUC5B
DHX30	LPA	PKHD1	SLC26A1	AASS	B9D2	MT-TR
RDH14	NFKB1	CSF3	KCNK1	THBS2	TSPO	HRH1
IQSEC1	LCN2	ROBO1	CHIT1	POC1B	HBG1	C1QBP
RNASE3	IL1R1	NOTCH2	SMAD2	WDR19	CASP12	CSNK2A2
C5AR1	FAM167A	IL13	PRODH	NOG	S100A13	DHODH
UGCG	MIF	MIR423	RNLS	COQ8A	KCNN3	RCN2
COX5B	ZDHHC24	USH2A-AS1	MIR29B1	MIR451A	GABRR1	UBB
LMBR1	ZFYVE26	CFAP410	EDN2	BANCR	KDM6A	PEBP1
COX6C	MAPK8	EMC1	LAMP1	OTC	STUM	AOX1
OSGEPL1	THBS1	STUB1	MATN4	CTSF	EGFEM1P	GATA5
MRPL18	MT-CO3	GAS1	SYVN1	BLOC1S1	LINC01646	STT3B
GPR22	FGF21	HNF4G	SKIV2L	SLC16A1	AURKA	NUP107
OR4L1	JAZF1	GPR35	NPHP4	SRF	ABCC9	DNAJB6
TRMT61B	G6PC1	OCA2	FA2H	LOC107303340	NFATC2	GBX2
YRDC	NPHP1	PCNT	TNFRSF13B	CROCC	NLRP12	SLC30A5
MRM2	KIF11	METRNL	NANOG	CTSG	PYGM	ZBTB18
MT-TD	SLC40A1	SGK1	IL9	IL33	EIF4EBP1	GTF2F1
TRL-AAG2-3	PRL	RAC1	GUSB	SLC39A14	OTX1	ADAMTS6
CYP4F11	WDPCP	PDE6B-AS1	KLF9-DT	LGALS1	EPRS1	AGAP1
BIRC2	CERKL	LOC122152296	IQCB1	ATXN1	HLTF	PIGL
GAB2	SOD3	LOC112806037	STAG2	RDH8	ASL	POP4
CD276	FN1	ATXN2	RUNX1	SDS	RING1	SLC28A3
MSLN	MT-TS2	NDUFV2	IL17F	FBL	GCDH	CCHCR1
SPG21	AIP	CELA3B	SLC4A4	PDCD1LG2	MIR138-1	PPP1R10
MYH2	PTGS2	COX15	TACO1	TFB1M	HSPB2	PSPN
FRZB	PVT1	LOC106099062	MITF	IL12RB2	CENPB	TSEN54
KLRC1	MEG3	NR1D1	NDP-AS1	YAP1	PTGES	SSR3
H3C1	FXN	ALOX12	NRG3	MAPKAPK3	PATJ	CMTM8
SMPD2	CPE	CHGA	SH2B1	KRT7	SLC7A5	ARHGEF28
TNFRSF12A	IMPDH1	AFG3L2	MYC	CAPN1	MIR22HG	OSBPL10
RHOD	FABP4	PSTPIP1	TRPV3	UNC119	CR2	TRIM58
ABCA2	MIR96	TTLL5	CNGA3	ATXN8OS	NPR1	PLD6
CDS1	RCVRN	LOC110006319	ABHD12	GGCX	FAM151A	GUCY1B1
DLL3	MT-TH	INPP5E	TYMP	CAPN2	CAMK4	SF3B5
MMAA	CYBA	TENM4	VCP	LAP3	EMX2	UBXN2A
GAB1	CCL5	HLA-C	AIF1	MAPKAP1	KIF21A	CASKIN2
CXCL11	FNDC5	CC2D2A	EDN3	PF4V1	OPA3	CNOT10
CYP4F12	HADHA	MFSD8	NPPC	NRAS	PISD	ZNF557
CD47	ALDH2	ELOVL5	IFT52	SCG2	IRF7	FAXC
FOXG1	PRCD	MT-TT	CHN2	PINK1	GPSM1	TBC1D19
OSBP	CNGA1	TDGF1	CPVL	NR2E1	FOXN4	PSORS1C2
KLHL1	IL2	HKDC1	ADCY3	PSAP	FCER2	ZNF572
SLC7A2	TOPORS	SERPINA3	PANK4	MIR30B	SRR	ZNF860
LINC02914	C4A	ENG	LIF	E2F1	CYP11B1	FOLH1B
MRPL12	ZNF513	TNFSF10	MTFMT	DDAH1	ABCA12	OC90
CDA	RP9	PKLR	ATP6V1A	PALS1	SLC6A3	OR13G1
RSAD2	PTX3	ACADS	SP3	TMSB4X	MRPS22	OR13D1
FGF18	OFD1	CD28	GRB2	FGF5	LRP8	OR2G3
KATNB1	KL	EPHB4	SDHAF2	ACVRL1	MIRLET7A1	PSORS1C1
IGSF3	FOS	GNRH1	TGFBR1	HELLS	RMI2	TMEM94
TUBB6	MERTK	MOG	ATP6AP2	MIR16-1	RAD54L	MYORG
HAPLN2	CAMK1D	TRPV1	HPRT1	KCNMA1	RECQL	C1orf94
SHE	TFR2	EPAS1	HTT	DNAJC5	DHFR	OR6N1
CEP97	IDH3B	CD40	C1R	ITGAE	MYBPC1	ZFP82
RALYL	MTOR	VHL	CEBPB	SEC23B	GRIN2A	SFTA2
PPP1R35	GFAP	MAPK14	MLX	CEP135	SUCLG1	OR2T8
DRC7	PRPF8	CSN2	TRPM6	ACTG1	TSPAN7	ST20
MTRNR2L5	HGSNAT	GLRX5	MGP	XPA	ERAP2	MUC22
COL6A3	G6PD	FCGR2A	VEGFB	DPP10	BSN	PSORS1C3
GRK6	TNFRSF1A	NDUFV1	GNB1	PCYT1A	GPR158	HCG22
KMT2D	CNR1	PTGDS	EXOSC4	SCAF8	TENM2	ZNF735
EXT1	ATRIP	COQ4	PGR	CNKSR3	MIR411	LINC00880
FOXE3	LRAT	SLC5A1	NES	TLR1	SCAF4	OR14L1P
H2AC4	CNGB1	TRIM37	ALPP	TNFSF15	SRGN	TIPARP-AS1
CCDC88A	ZNF408	CNBP	NDUFB8	PHACTR2	CLN8	DPP10-AS1
CA3	GPX1	MORC2	TNFSF12	FECH	TNPO1	LINC00536
PDYN	RBPJ	NF1	MYO5A	C5AR2	TBCE	LINC00881
UPB1	RP1	MIR203A	PSMA5	CD200	CLUAP1	ARHGAP22-IT1
MEIS1	GUCY2D	HMGA2	ACOX1	COL5A1	LSM8	LINC01539
EGFL7	PRKCD	MT-TI	NMNAT1	F2RL1	ARL13A	LINC01844
APOBEC1	FAM161A	MIR20B	C5	TRAF2	POTEF	LOC285626
CCL7	LZTFL1	FOXRED1	FN3K	DCX	MICU1	LINC01905
SLC22A23	CDH23	NTRK1	PET100	ALAD	BCO1	THRAP3P1
C1QTNF2	BMP6	PALB2	TGM2	CGAS	PHKA2	LINC01845
FAM98C	ACE2	CX3CR1	PLAUR	PIM1	BDNF-AS	LINC01947
PTGES3	SHH	IGF2-AS	COX4I1	ACP5	POLR2L	RPL13P12
XPO1	KIZ	CLRN1-AS1	EIF2AK4	ITPR1	PLK1	BALR6
TDP1	AMBP	ABCC6	HPE6	H2AC20	FGFR3	LINC02090
RASSF1	PCARE	NOX4	HPE8	ROCK2	TSC22D3	MIR6891
P2RX5	CAPN5	NDUFS7	HSD17B10	KMT5B	ACAD11	PRDX6-AS1
TGM6	RP1L1	HTRA1	PTGDR	TPH1	TRNT1	RPL31P12
TWSG1	PRSS23	MICA	ABHD11	CALB2	MAP2K7	STK19B
ZIC5	GNB3	CFI	OPTN	TMEM237	POU4F1	LINC02511
PUS10	FOXA2	NDUFS1	KIF3B	BACE1	RBM17	LINC02163
EPHA4	IGFBP2	BAMBI	TNFAIP3	MB	GRM1	LINC02349
PJVK	TERT	PKD1	DKK1	HYOU1	GRK2	ENSG00000241770
CHERP	ANGPTL8	RNU4ATAC	VPS13C	CETN3	MYO6	ENSG00000243176
SHOX	NOD2	TSPAN12	TRH	SYN3	ATP8B1	ENSG00000250237
PXN	IFT27	PAX2	FANCA	PARN	SRRM1	ENSG00000272501
CPOX	ARL3	MFN2	IL27	BCL2L1	PAFAH2	ENSG00000261757
TCHP	LEPQTL1	EGR1	VEPH1	TGM1	TNFRSF9	KRT8P39
PIERCE1	PTCH1	VSX2	LAMP2	SUGCT	HSD17B4	OR13D3P
TBX2	SPP1	P3H2	MIR146B	GCH1	OTOF	OR3D1P
DHPS	EGF	ADGRA3	CX3CL1	MIR149	PDHX	RNU1-58P
EHBP1	MED12	RP22	MT-ND4L	CPM	IBSP	RNU7-4P
AGFG1	FSCN2	RP24	DHX16	STAT2	NRTN	RPLP0P9
CCNH	ARHGEF18	RP63	MAOA	GALK1	BACE2	RNU6-667P
KAT6B	CRYAA	RP29	MIR15B	RARB	RAD51	RN7SKP230
JAG2	AHSG	RP34	COX5A	MYB	GP5	RPL26P11
CTTN	CYP19A1	PPP1R3B	COL18A1	LOC110806263	NTN4	TPT1P4
RDH13	NGF	TERC	DDX58	MIR152	CRYM	BDH2P1
H2BC3	CDKN2B-AS1	PTGS1	C19orf12	NEFL	DUSP1	MAN2A1-DT
ACVR1C	CDKN3	KLKB1	COL4A4	MT-TG	ATN1	LINC02672
POLR2I	RGR	PKD2	SCG3	CTCF	TRIM73	LINC02196
ITM2B	TTR	ITGA4	S100A12	RAX2	ACVR1	LINC02319
SNCG	PDE6B	IL2RB	GATAD1	MAP3K5	NAALADL2	LINC02814
GPR20	PRKAA2	COMT	EFTUD2	JPH3	XRCC5	ENSG00000248359
PLN	MT-TW	CYB5R4	S100A4	NPR3	ECM1	ENSG00000265511
GALNS	KNG1	RCBTB1	ENPP2	INSL3	RBL1	ENSG00000235749
IL15RA	VPS13B	VANGL2	GUCA1A	UBE2D2	PSMD1	RNA5SP146
MSX1	AIPL1	MIR93	RHD	KIF17	LAMB1	RNU6-169P
FUCA2	PTPRN2	PDGFRB	MAX	FADD	WNK4	RPSAP37
PIK3AP1	GJB2	ERN1	KIF1B	KRT19	SERAC1	LRRC77P
LDHD	MT-ATP8	DMPK	IDS	CYP11A1	WWOX	PPP1R2P6
PUF60	TAB2	GADD45G	DMD	SERPINB1	MIR431	ENSG00000272221
ADGRG1	IGF2R	AKR1B10	PRKCG	CDH17	MIR1281	ENSG00000274840
FUT4	CLRN1	RHOA	MIR200A	IL26	DYNLT3	ENSG00000223872
WDR7	IFT140	ITGB2	KRT18P34	PFAS	PSMD12	UBA52P7
EPHB3	POMGNT1	PARK7	MIR150	SQSTM1	VIPR2	LOC107985164
KLHL10	HSPA4	HLA-DMB	ACO1	KCNJ13	NDUFC2	ENSG00000233191
ACOXL	CFH	TFAM	HSPA1A	TREM1	PFKFB3	ENSG00000272540
CCND2-AS1	ANGPT1	SIRT3	CGA	POT1	YWHAE	RNA5SP459
MIR8085	GAST	XDH	WDR45	UCA1	EGFL8	SRSF6P2
NEDD8	F3	AGTR2	LINC01611	AMPD1	ITGA3	AMD1P1
COQ8B	SLC12A3	C2CD4B	AQP8	KLRK1	CDK7	ACTG1P24
SEPTIN11	PCSK1	FMR1	MICB	PITPNM1	H2AX	RNU4-77P
SMARCA2	MMP3	MT-TP	LIPA	ATF3	OGDH	RNU6-938P
ATP1A3	GDF15	HLA-DMA	NEK8	TKTL1	ELAVL3	ENSG00000271581
YARS1	ATRIP-TREX1	SUFU	FUS	ULK1	ELAVL2	ENSG00000225311
STAB1	UTS2	NOTCH1	MYT1L	RDH10	RECQL4	ENSG00000241596
SLC6A11	GUCA1B	ERCC5	NFKBIL1	NUTF2	IHH	ENSG00000255429
ANAPC2	APOA4	AKT3	PLAU	CLCN2	ARSA	ENSG00000283584
RAX	FABP1	LRP2	NDUFA1	FSTL1	GRIA2	ENSG00000287340
PCGF1	KIF7	MAPT	NDUFB3	FBXL4	KLHL3	HNRNPA1P46
PSD4	PRPF3	TJP1	PON3	ALDH1A1	ORM1	RN7SKP15
INTS12	HERC2	MIR22	SNHG6	TP53BP1	AHCY	RN7SL691P
ARHGEF38	DHX38	PEX10	GHRH	MMRN1	TOP1	NONHSAG043472.2
CTSA	GSR	PIWIL4	SETD2	SLC25A20	TRAPPC10	lnc-FRMPD2-5
GRID2	CREB1	COL4A5	GRAMD2B	STING1	SLC13A5	ENSG00000216475
RPS6KA2	ELN	CEP41	CDK6	RARA	GPAT2	lnc-LEKR1-42
FUT9	B2M	EFEMP1	DCN	DLST	SCP2	lnc-SF3B5-3
ZNF141	RPGRIP1	NKX2-2	DGUOK	MAPRE2	OPN1SW	lnc-LEKR1-6
LTO1	CTNS-AS1	CFB	MVK	IL12A-AS1	NYX	lnc-RHEB-2
HGS	SNRNP200	APP	LIG1	CLSTN1	ATP5PO	lnc-PIK3R1-10
SEPTIN5	AMACR	S100B	STN1	CLDN2	FTX	MK280144-591
SEPTIN8	GSTP1	CCND1	NTRK2	LIFR	MBTPS1	lnc-HLA-C-2
DKK2	AHR	ADAR	MTRR	DDB1	DLGAP1	lnc-CCNL1-4
COL6A2	PECAM1	CALR	CRYGS	CASC2	EPHB2	lnc-CCNL1-3
H4C15	NR1H2	NDUFS8	EXOSC2	SLC25A37	PNKP	piR-47864
KAT2A	SNRPN	MT-TA	FASTKD2	COLEC12	GLIS2	piR-38220
ATXN7L3B	SELL	MIR204	RXRG	ZNRF1	CYP2B6	HSALNG0024535
HSFX1	IDH3A	KLHDC7A	PRF1	GORAB	CEP250	HSALNG0030090
GNMT	CA4	SERPINA4	TIMP4	API5	FLCN	HSALNG0049258
ALDH3A1	LCA5	SLPI	TRIM28	TULP3	ARF3	HSALNG0054066
ELAVL4	SOCS3	CHEK2	EZH2	SMARCA4	TULP2	HSALNG0054232
PLPP5	BLM	IARS2	ALDOA	HDAC1	CAMSAP3	HSALNG0028211
KIF2A	PCK1	CSF2	TRMU	MIR200C	SRA1	HSALNG0052169
ESX1	LCAT	TWIST1	DARS2	RORA	GPHA2	HSALNG0030098
H4C2	TNFRSF1B	KCNQ1OT1	RNASEH1	ARR3	HAX1	HSALNG0123625
H4C3	NLRP3	STIL	SLC34A1	SUV39H1	INHA	HSALNG0007431
H4C12	PAPPA	GLUL	OCRL	PITPNC1	ALCAM	HSALNG0008504
H4C8	ERCC6	NEAT1	FARSB	PTGER3	SLC45A2	LOC100420048
H4C5	SERPINC1	PEX7	GPR161	PALM2AKAP2	LCOR	lnc-UTP23-5
H4C11	ASTN2	SYP	HSP90B1	MSH5	HTR2B	lnc-VEPH1-1
H4C14	CYP27B1	CANX	NDUFA12	LY6G5B	PHF6	MK280073-024
H4C9	PEX6	EPOR	NDUFA9	LOC106627981	MIR2116	MN298114-181
H4C13	FLVCR1	POU5F1	NDUFA2	NFIB	MIR3197	MK280269-056
H4C6	TXNIP	SPARC	TRAF3IP1	APOA1-AS	CCNA2	HSALNG0007430
H4C4	MIR27A	COL4A1	SULT1A3	OAT	HPR	lnc-IL12B-2
WNT16	SOX2	TNFRSF11A	PCNA	XRCC2	NPR2	ENSG00000287114
APOBEC3G	PRPS1	CD59	LSM2	PTK2B	FGF3	HSALNG0123626
TCTN1	IL6R	ELMO1	CABP4	SLC25A46	WNT10B	HSALNG0103761
ADAM15	FADS1	CTSD	ADGRV1	BHMT	AGO2	lnc-CCDC125-21
MIR19B2	KIAA1549	BRIP1	GAP43	TBXAS1	ATOH7	piR-52740
GCM1	ERCC8	CD38	KLHL22	SSTR1	CRLF1	RF00017-3200
EDIL3	AGBL5	MIAT	GRIK2	F9	LUC7L2	RF00017-6351
ETFA	REEP6	MYSM1	UGT1A1	NCOR1	SNU13	RF00017-6352
GATD3	PLA2G7	HESX1	FOXP2	IL11	KPNB1	piR-54121
MYO3B	MALAT1	RDH11	MIR26A1	MIR124-1	GLI3	RF00017-2562
CD27	CDK4	HDAC9	HCRT	MIR137	CELF1	piR-56310
PITX1	CYP2C9	LGR5	TNC	MGME1	FLNB	LOC102724446
POLR2B	BRAF	GHSR	IFNB1	CLDN1	BBOX1	piR-32810–107
WSCD1	COG2	MNX1	LTF	STX3	SFRP2	piR-39341–315
MIR410	ERCC4	KIFC2	EHHADH	HIRA	PDK1	piR-48965–070
S1PR2	TUB	VPS18	SNCB	MRPS27	ZRS	MIR1-1
ABCB6	TAS1R3	PRDM15	UBQLN2	LIPH	CCT2	MIR124-3
PSPH	RAPGEF3	WASHC5	RAD51C	METTL3	BMPER	PSMD14
STX1B	KIF5B	IGBP1P1	MTMR4	FAR2	SLC30A1	CUL7
DYNLL2	EVC2	LOC100507346	SEC24B	DYNC2I1	DUSP19	PPM1E
SIX4	PMEL	SBE2	MLANA	INPP5B	ADSL	NFE2
KLHDC2	TMEM138	CASK	TRIM23	MCCC1	CDK5R2	FGF14
SEC14L3	CDH16	GP9	HOGA1	HBD	ADAMTS19	WIF1
DYNC1LI2	EXTL3	RASGRP2	TRIM17	NME3	UCKL1	EAPP
MICAL3	CDK13	MSH6	TRAT1	PORCN	STON1	BHLHE41
FAM53B	ZFR	RIPK2	SKA2	GUF1	RIPPLY1	COQ7
HSPB11	MIR543	SEC24C	LACC1	ASB7	PSMA7	MRTFA
FBXL15	USP14	SLC17A1	PNPLA7	TM2D3	MDM1	ARAF
ACSM4	PLP1	PTCRA	PRR11	AFDN	MFF-DT	HAO1
IFT46	TUFM	PNPT1	EYA4	TECPR2	LIM2	UBE2D3
TAS2R46	SLC1A6	PLAG1	RAD21	CPLANE1	PTBP1	GOLGB1
HEATR5A	GNPTG	MYH11	FBXW11	CYP51A1	CXCL13	GLRX
DYNC2I2	LGR4	GAMT	PRRX1	GTF2H1	SMARCC2	SEC31A
FAM186A	MAPKAPK2	POLRMT	BOC	ADAM28	SULT1A1	SIM2
DYNLT2B	RPSA	SLC8A3	TGIF2	TYR	SNHG16	KAZALD1
CWF19L2	NRP2	CAND1	ZIC4	SOX10	TFG	ZRSR2
RO60	DLD	PLPPR5	WDR81	PSMD7	PLPP3	H2AZ1
MARCHF3	PPIB	FAM170B	SLX4	CCDC96	PIANP	GRIN2D
VWA3A	CDKN2C	DYNLT4	AKAP4	CCDC172	MRPL36	SNX13
ASAH2	IRF4	PRAMEF12	ADCY4	CFAP97D1	ATP5F1E	VWA1
ELOC	ALG2	AHSA2P	RAB10	ABCA3	UBXN7	PROM2
MIR18B	ARSH	SNORD42B	ICAM2	POLE	DPH3	CLCN7
RFXANK	ELOB	MIR210HG	RPS9	PIKFYVE	DENND4B	PDHB
TLE2	TSPAN16	MIR651	SLIRP	MLF1	PLAAT1	NFE2L1
GRIN3B	ARID2	PRR21	DCT	ADAM23	HLA-DPB2	HIBCH
MTF2	SSB	ENTPD5	EIF3A	DCTD	GATA6-AS1	CDH8
SOX1	MARCHF6	FGF16	SLC22A8	NLGN4X	LOC112081413	KDM4A
UQCC2	MRPS12	GMFB	SEC13	GTF2H4	COL6A1	UQCRQ
ZNF71	APLP2	VASH2	SLC34A3	NEIL2	RPS23	THEM4
UNC93B1	ANXA3	MIR5195	LMBRD1	GTF2H2	CEP128	CBX7
CD160	CCT4	CD151	FHOD1	GTF2H3	ADCY9	CAPN8
GDE1	APOBEC3F	PTDSS1	HSCB	SSH3	DPEP1	VPS26B
AP3D1	TEAD1	UBR2	PLLP	MAN2C1	RPS13	PYCR3
SLC8B1	CLCF1	WDR73	GLYATL1	UNC80	SCAI	FOXO6
DEPP1	STIP1	AGGF1	EGLN3	GYPE	IFNA21	TUFMP1
GNAI3	DENND4C	PIP5KL1	SLC18A1	DVL1	JOSD2	PLCG2
FBLN2	MAP2K3	SLC6A15	DAO	GPR146	PTPMT1	MSRB1
ALDH5A1	TUBB2A	RNASE1	CDH15	CA7	MZF1	MOAP1
RGS5	SLC7A7	RGS16	FBXO7	AKAP12	ALDH16A1	TPTEP1
GSDMD	ACTR2	AP1S3	CHIA	AFG3L1P	SIAH1	SUPV3L1
HSP90AA2P	SEMA3F	TLE6	UCHL5	HOXB3	PTPRB	ENTPD7
MIR646	HPS1	BPIFA2	SLC35C1	PNPO	COTL1	PREX2
ENO3	C10orf88	DYNC1H1	TTC19	EGLN2	SERPINB9	ELF3
ST3GAL5	OR52B4	UBASH3B				
Pyroptosis						
FADD	GSDMD	APIP	GAS5	TFAM	MIR497	FNDC4
VCAM1	GSDME	IL18	TP53	PGF	EPHA2	FNDC5
SESN2	NLRP3	HMGB1	VDR	NLRX1	ABL1	ELANE
TNF	CASP1	STAT3	PCSK9	SLC16A4	HDAC6	PARP1
VIM	GSDMC	MALAT1	BRD4	IL32	SQSTM1	TRIM21
CAPN1	GSDMB	SIRT1	IKBKE	CHRFAM7A	IRF3	PRKN
JUN	CASP4	KCNQ1OT1	AGER	MIR21	CDK9	GBP5
RIPK3	NLRP1	TREM2	PKM	MIR124-1	TREM1	NR1H2
MIR139	GSDMA	MIR223	CRTAC1	MIR195	TSLP	CTSG
BHLHE40	GZMB	FOXO3	TET2	MIR485	ZDHHC1	MKI67
BHLHE41	CARD8	MIR30C1	CTSV	MALT1	STING1	IL36G
ALK	CASP8	NFE2L2	UTS2	TLR2	HNP1	IL36B
TFAP2A	GZMA	NEK7	MIR155	GSK3B	PTEN	NLRP6
BIRC3	IL1B	TXNIP	MLKL	STK4	DRD2	PRTN3
E2F4	DPP9	DDX3X	NFKB1	PTGS2	ADORA1	SERPINB1
BIRC2	DPP8	MIR22	APOE	MST1	ADORA2B	DUOX1
UBE2D2	NLRC4	MIR125A	SDHB	PRF1	ADORA2A	APOL1
LY96	AIM2	GBP1	P2RX7	TRIM24	ADORA3	MEFV
GLMN	CASP5	MEG3	EEF2K	ELAVL1	METTL3	FOXP3
IRGM	ZBP1	MIR135B	CD274	MPEG1	PECAM1	NLRP7
SCAF11	PYCARD	MIR556	FGF21	MIR204	TRIM31	ANO6
NLRP13	CASP3	GJA1	DLX6-AS1	HOTTIP	METTL14	BNIP3
ADAMTS9-AS2	NAIP	UBR2	MIR23A	CDKN2B-AS1	MIR25	XIST
NINJ1	CASP6	CPTP	KLF3-AS1	MIR9-1	IFI16	MIR107
TUBB6	DHX9	PRDM1	CEBPB	MIR9-2	CAMP	MIR103A2
MYD88	NLRP9	MIR214	BSG	MIR9-3	MRE11	MIR103A1
GPER1	TLR8	MDM2	TNFSF13B	IRF1	YWHAZ	TRPM2
BST2	APAF1	BTK	BECN1	ATF6	STXBP2	CHMP4B
LYST	NOS1	BCL2	CHI3L1	CASP9	UBE2D3	PDCD6IP
VPS28	NOS2	IL1RN	RAB5A	IRF2	TLR9	VPS4B
NCR1	PKN2	RIPK1	PANX1	ORMDL3	CD14	ATG3
IL27	DPEP1	YWHAE	IL13	POP1	IFIH1	CXCL8
SEC22B	CHMP1A	HSP90AA1	ASIC1	LINC00958	GSTO1	IL13RA2
SIGLEC14	PYDC2	ANXA2	BRCC3	MIR4306	HUWE1	STXBP3
CLEC5A	ACE2	NEDD4	ATG7	ERP44	IRAK3	EGFR
CGAS	AKT1	HSP90AB1	LRPPRC	CDC37	MELK	TP63
MIR20B	MIR15A					
SAL						
MIR27A	MTOR	CASP8	NFKB1	NLRP3	CDKN1B	HIF1A
MIR138-1	GSK3B	PCNA	TLR4	KLF4	NOS3	LOXL2
ALOX5	SIRT1	CASP3	NFE2L2	PRKN	PREP	

### GO and KEGG analysis

**Fig. 5:**
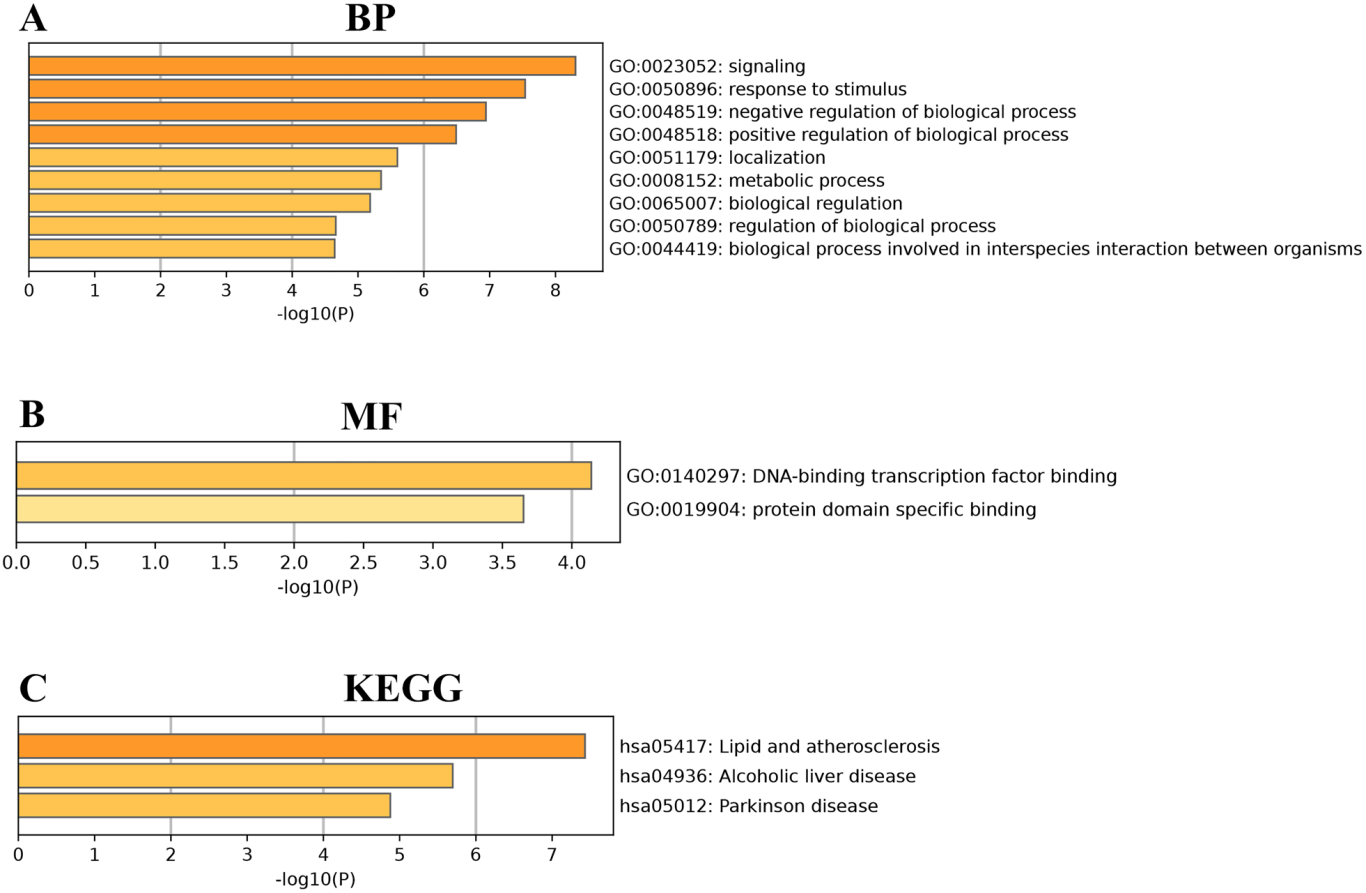
GO and KEGG pathway analysis

The intersection genes were imported into to Metascape database to perform GO and KEGG analysis, we selected custom analysis, and selected "BP", "CC", "MF", "KEGG" in "Enrichment" for detail analysis. Only the biological process (BP) is enriched in the GO analysis. The first 9 pathways are: Signaling, response to stimulus, negative regulation of biological process, positive regulation of biological process, localization, metabolic process, biological regulation, regulation of biological process, biological process involved in interspecies interaction between organisms. In addition, two molecular functions (MF) were acquired which includes DNA-binding transcription factor binding, protein domain specific binding. At last, KEGG signaling pathways are pointed Lipid and atherosclerosis, Alcoholic liver disease, Parkinson disease (Fig. 5).

### Construction of PPI network and screening of Hub genes

**Fig. 6:**
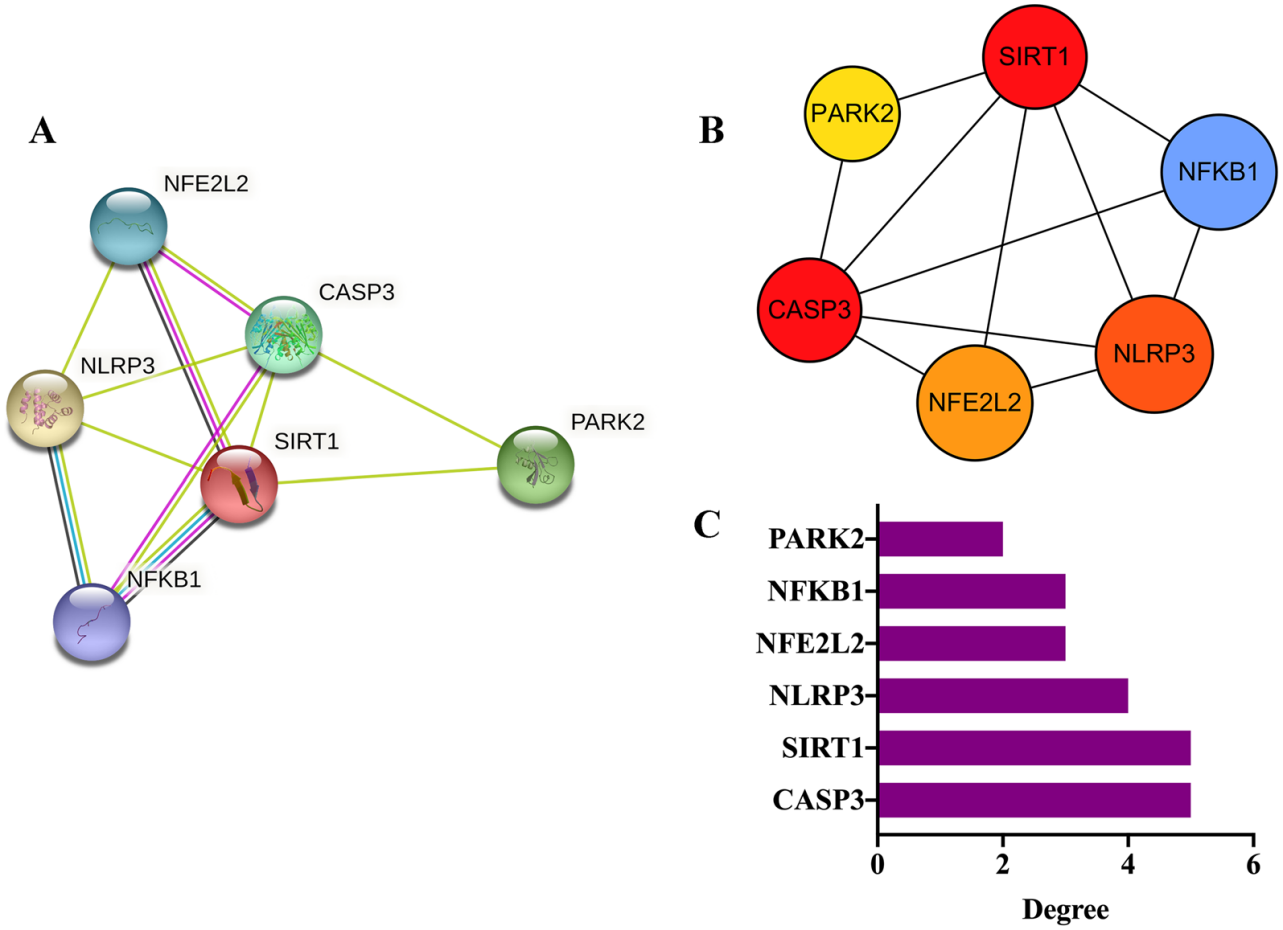
Construction of PPI network and screening of Hub genes. **A** Interaction network of intersecting targets. **B** Screening of hub genes. **C** The degree value of hub genes

In the STRING, the website is: https://cn.string-db.org/, and the interaction between genes is analyzed by using the intersection genes. It can be seen that an interaction relationship exists among 6 genes. According to the degree value, the Hub genes are screened and visualized, and sorted according to the degree value from large to small, in order: CASP3, SIRT1, NLRP3, NFE2L2, NFKB1, PARK2. (Fig. 6B, C).

### Molecular docking verification

**Fig. 7:**
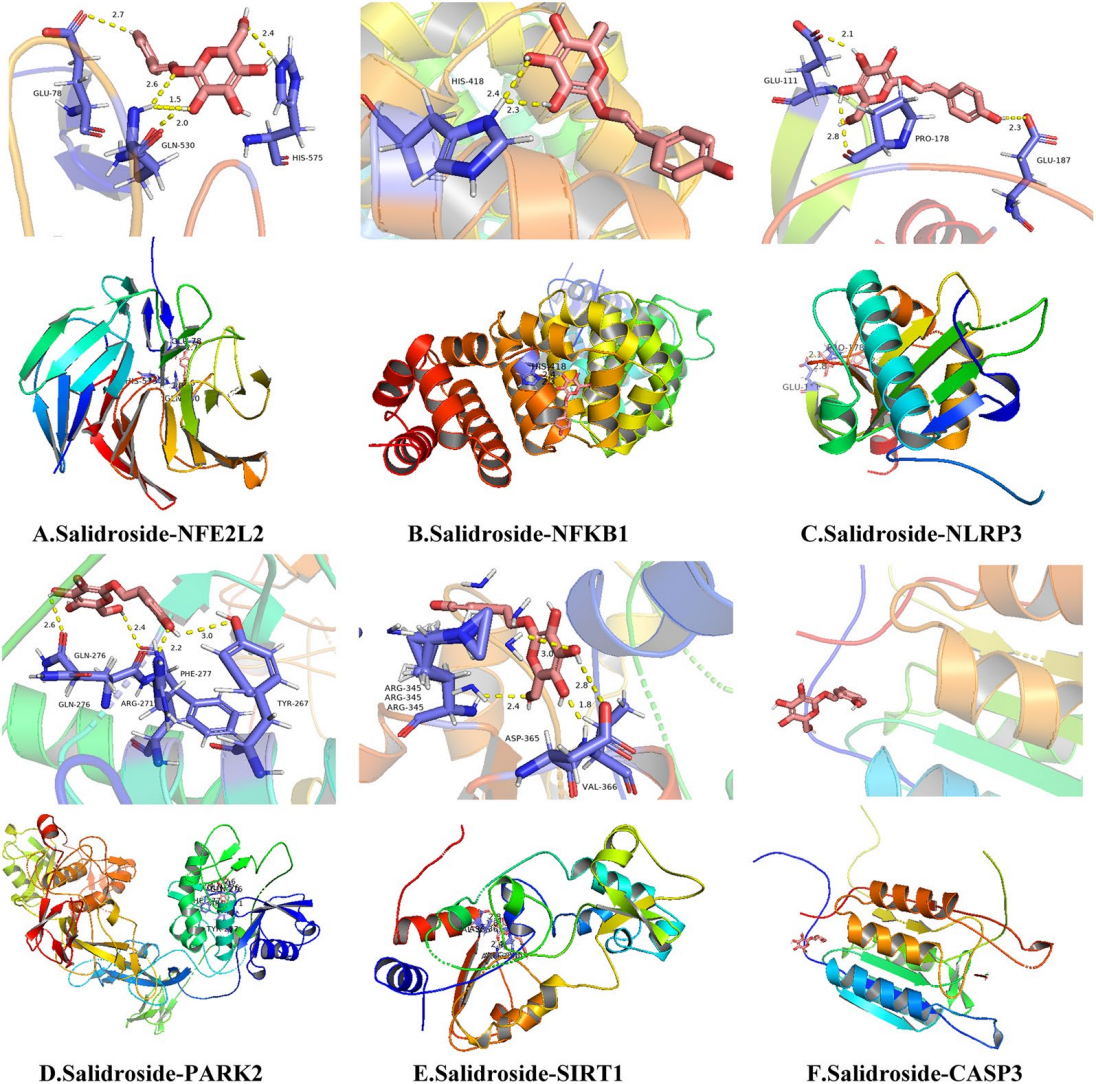
Molecular docking verification. **A** Salidroside-NFE2L2. **B** Salidroside-NFKB1. **C** Salidroside-NLRP3. **D** Salidroside-PARK2. **E** Salidroside-SIRT1. **F** Salidroside-CASP3

The screened core genes were imported into PDB (https://www.rcsb.org/) to query the 2D structure of the protein. Meawhile,the chemical structures of drugs were searched in the Pubchem database (https://pubchem.ncbi.nlm.nih.gov/). Then, molecular docking was performed with Autodock software. Consequently, SIRT1, NLRP3, NFE2L2, NFKB1, and PARK2 can form stable molecular structures with salidroside, while CASP3 cannot dock with salidroside. Through molecular docking verification, we speculated that NFE2L2, NFKB1, NLRP3, PARK2, and SIRT1 genes have regulatory effects on the pyroptosis of RGC, so as that they, could be considered as possible target for salidroside to prevent diabetic retinal damage (Fig. 7).

### qRT-PCR

**Fig. 8:**
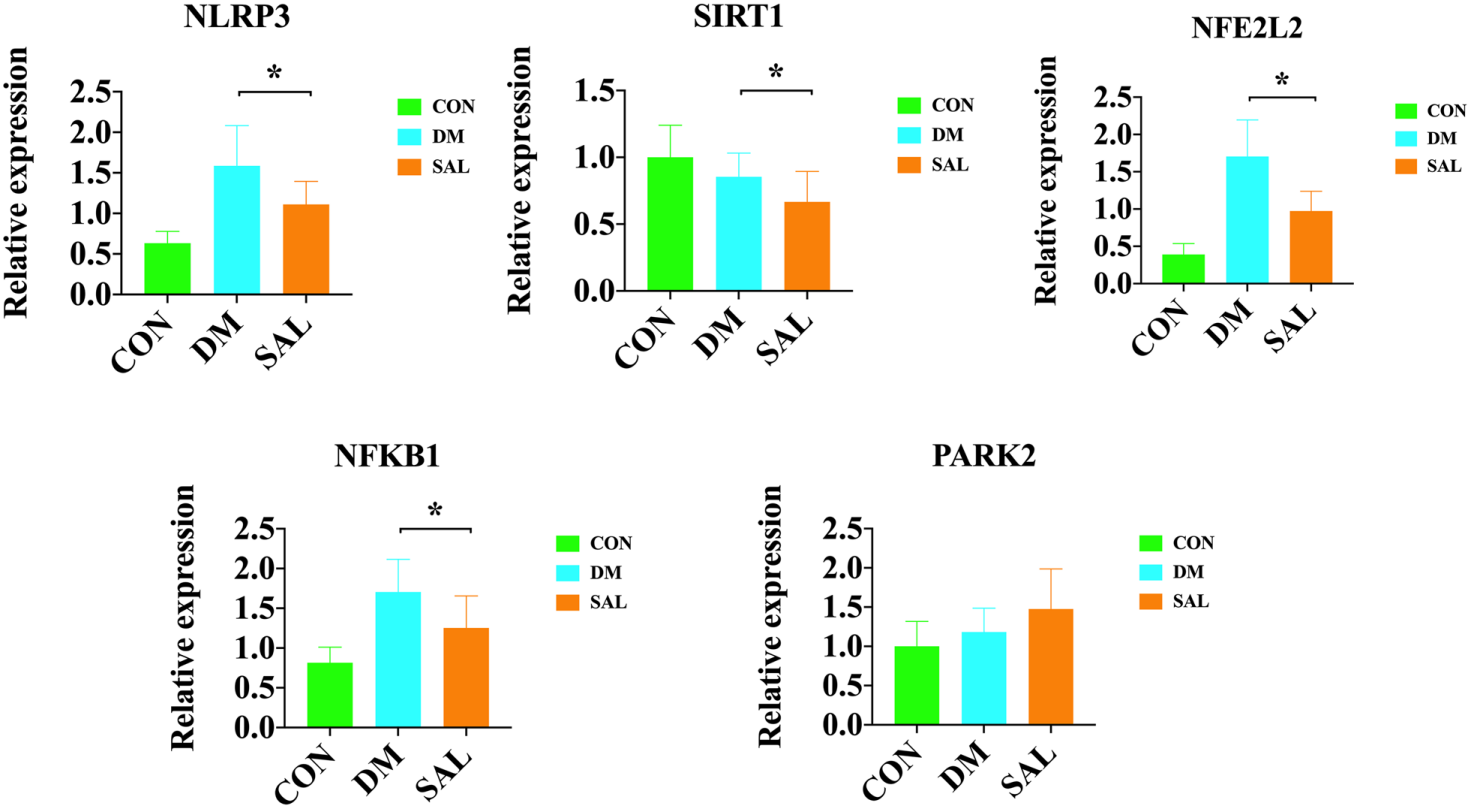
The results of qRT-PCR. (From left to right, from top to bottom represent the relative expression of NLRP3, SIRT1, NFE2L2, NFKB1, PARK2 in CON, DM, SAL group)

Compared with the normal control group, the mRNA levels of NLRP3, NFE2L2, and NFKB1 in the diabetes model group were significantly increased, while they decreased in the SAL treatment group (*P* < 0.05), with the statistical difference. Differently, the mRNA level of SIRT1 in the diabetes model group was lower compared with the normal control group, and it got the lowest level with statistic significant, when compared with in DM group. Lastly,, the mRNA level of PARK2 in the diabetes model group increased, but there is no effect after SAL treatment (Fig. 8).

## Discussion

In this study, we found that the retinal layers in the CON group were the most complete and neatly arranged, with normal cell morphology, clearly visible internal limiting membrane, and ganglion cells arranged in a single layer. Comparatively, in the DM group, the number of ganglion cells gradually decreased with obvious edema increased, and the distribution was disordered and sparse. Whereas, in the SAL group, the edema of cells in each layer was significantly reduced, the arrangement tended to be regular. In addition, the fluorescence staining showed in DM group, the intensities of marker proteins were all the strongest and the positive value was high, while they decreased and became moderate in the SAL group, which is different from the lowest fluorescence in the CON group. In network mechanisms, pharmacology network analysis acquired 6 genes by venny intersecting, and the results of PPI analysis showed that there was a close relationship among 6 genes, and the NLRP3 gene had the highest comprehensive score. The verification of molecular docking showed that CASP3, other NFE2L2, NFKB1, NLRP3, PARK2 and SIRT1 could combine with salidroside, and qPCR verified the change of NLRP3, NFEZL2 and NFKB1. Our results reported that SAL inhibited effectively diabetic retinal RGC pyroptosis, which is associated with NLRP3, NFEZL2 and NFKB1 and multiple pathways, indicating that SAL could be considered as a potential drug to treat and protect DR, and underlying network pharmacological mechanism is involving in anti-inflammatory signal pathway.

### HE and Nissl staining

In this study, HE and Nissl staining showed that the retinal layers of the CON group were the most complete and neatly arranged, and ganglion cells arranged in a single layer, while the retinal cells of the diabetic group were disordered, the ganglion cell layer and the inner and outer nuclear layers had obvious vacuolar degeneration. Comparatively the addition of SAL reversed these changes. These suggested that SAL could effectively inhibit retinal thinning, reduce cell damage and neuron loss to improve DR damage. It has been reported that (Ji et al. [8]) the retinal tissue of normal control rats did not have pathological changes, while in the diabetic model rats, it showed the significant retinal changes. One study showed that [30] morphology of the retinal cell did not obviously change after 4 w in DM group, nevertheless, retinal thickness was significantly thinner and RGC numbers were significantly reduced at 4 w. What’s more, it also presented retinal cell of INL and ONL arrangement disorder. Moreover, some studies have found that SAL had an inhibitory effect on nerve damage caused by many diseases [7] and SAL inhibited the glutamate-induced apoptosis of rat hippocampal neurons [32]. Here, we confirmed the effect of SAL in the improvement of morphological character in retina of DM,, which is useful to the usage of SAL in clinic.

### Immunofluorescence analysis

In our study, the fluorescence of DM group for observed genes was the strongest, but the addition of SAL lowered fluorescence intensities. Yin et al. [37] observed that NLRP3 and CAP1 were localized in the RGC layer and INL by using immunohistochemistry. At the same time, they also found that the expressions of CASP1, NLRP3, and their downstream mature molecules IL-18 and IL-1B were increased in the retina of DM rats. Moreover, it has been reported that salidroside inhibited NLRP3-dependent pyroptosis in different disease [1, 2, 33], while there was not a research related SAL to ameliorate DR by inhibiting pyroptosis in previous study. Our findings provided new evidences to understand the effect of SAL in anti-pyroptosis of RGC after DM.

### Intersection genes and construction of PPI network

In this study, six intersecting genes were obtained by venny, and the results of PPI analysis showed that there was a close relationship among the six genes, and the NLRP3 gene had the highest comprehensive score. Previously, it has been known that the sirtuin (SIRT) family were involved in the development of various diseases such as neurodegeneration, cardiovascular pathologies, metabolic disorders, and cancer. SIRT1, 3, 5, and 6 were key enzymes in DR since they modulated glucose metabolism, insulin sensitivity, and inflammation [29]. Comparatively, in the present study, we showed that [34] NFE2L2 was an important component of the intracellular antioxidant machinery, in which, NFE2L2 could be considered as a target for treatment of diabetic complications. NF-KB is a nuclear transcription factor that can regulate the expression of various genes in inflammatory response, immune response, cell proliferation and apoptosis. The continuous activation of NF-KB increases the release of inflammatory factors in the inflammatory response, and regulates cell proliferation and apoptosis [22]. Numerous studies have demonstrated that the NLRP3 inflammasome plays an important role in the pathogenesis of various diseases [6], NLRP3 inflammasome retina caused by early hyperglycemia, and affecting the structure and function of the blood-retinal barrier [24]. Our results suggested that NLRP3 inflammasome and related proteins have been involved in the process of SAL administration after DR.

### GO and KEGG analysis

In this study, we found that the first 9 pathways of BP were enriched. There were two MF that is DNA-binding transcription factor binding, protein domain specific binding. Whereas, KEGG signaling pathways are: Lipid and atherosclerosis, Alcoholic liver disease, Parkinson disease. Multiple genes network analysis to uncover the mechanism of drug action is a hotspot based on bioinformatics and network pharmacology [31, 40]. Recent research showed that [20] SAL flattened high glucose-induced injury in retinal pigment epithelium cells by activating PI3K/AKT and AMPK signaling pathways. What’s more, SAL could suppress the P2X7/NF-KB/NLRP3-mediated pyroptosis [2]. In addition, SAL could decrease the expression of TLR4, NF-κB, P-NF-κB, NLRP3, ASC, cleaved Caspase-1, cleaved GSDMD, IL-1β, and IL-18 by inhibiting TLR4/NF-κB/NLRP3/Caspase-1 signaling pathway [1]. Together, all results showed that SAL may regulate DR to inhibit pyroptosis via various signaling pathways combined with our findings.

### Molecular docking and qRT-PCR validation

In our study, the verification of molecular docking showed that except CASP3, other NFE2L2, NFKB1, NLRP3, PARK2 and SIRT1 could combine with SAL, and qRT-PCR confimed the change of mRNA levels for NLRP3, NFE2L2, PARK2 and NFKB1.These results suggested that NFE2L2, NFKB1, NLRP3, PARK2 and SIRT1 might be proposed as new therapeutics to treat DR. Mortuza et al. showed that the expression of SIRT1 was decreased in human retinal endothelial cells with high glucose concentration [18]. Similarly, in retinal endothelial cells, hyperglycemia determines SIRT1 down regulation followed by a decrease of mitochondrial antioxidant enzymes levels through pathways controlled by p300 and Fork head box protein O1 [42]. These evidences pointed that SIRT1 was increased in DM groups. Comparatively, NFE2L2 had pivotal roles in many signaling pathways that were altered in the retina in diabetes, and were implicated in the development of DR [12]. Luo et al. [16] found that NFE2L2 were decreased in blood samples of DR patients and high glucose-treated human RPE and ARPE-19 cells. Whereas, the overexpression of NFE2L2 promoted proliferation and suppressed apoptosis and inflammation. This was exactly the opposite of the results of this experiment, which may be caused by different model condition, needing to be verified in later experiment.

Although, previous NF-KB studies have found that NLRP3 inflammasome was involved in the formation of pathological retinal neovascularization by establishing advanced DR animal models, it is lack evidence to show the relation between SAL and NLRP3 [3, 24].

Our results are the first time to show the effect of SAL in DR, which is associated with several molecular network that has been reported previously [9, 11, 28].

## Conclusion

The main novel findings are that salidroside can significantly improve the morphological retinopathy in diabetic rats, especially for RGC, in which, the underlying mechanism is related to the regulation of (NLRP3, GSDMD, Caspase-1, IL-1β, IL-18, Of them, NLRP3, NFE2L2 and NFKB1 could be considered as the direct target of SA, so as to provide the protection for RGC in our experimental condition.

## Data Availability

The data used to support this article are included within the article.
